# IL-21 drives expansion and plasma cell differentiation of autoreactive CD11c^hi^T-bet^+^ B cells in SLE

**DOI:** 10.1038/s41467-018-03750-7

**Published:** 2018-05-01

**Authors:** Shu Wang, Jingya Wang, Varsha Kumar, Jodi L. Karnell, Brian Naiman, Phillip S. Gross, Saifur Rahman, Kamelia Zerrouki, Richard Hanna, Christopher Morehouse, Nicholas Holoweckyj, Hao Liu, Autoimmunity Molecular Medicine Team, Kerry Casey, Michael Smith, Melissa Parker, Natalie White, Jeffrey Riggs, Beth Ward, Geetha Bhat, Bhargavi Rajan, Rebecca Grady, Chris Groves, Zerai Manna, Raphaela Goldbach-Mansky, Sarfaraz Hasni, Richard Siegel, Miguel Sanjuan, Katie Streicher, Michael P. Cancro, Roland Kolbeck, Rachel Ettinger

**Affiliations:** 1 Respiratory, Inflammation, and Autoimmunity Group, MedImmune LLC, Gaithersburg, MD 20878 USA; 2 Translational Medicine, MedImmune LLC, Gaithersburg, MD 20878 USA; 3 The Office of Clinical Director, National Institute of Arthritis and Musculoskeletal and Skin Diseases, National Institutes of Health, Bethesda, MD 20892 USA; 4 National Institute of Allergy and Infectious Diseases, National Institutes of Health, Bethesda, MD 20892 USA; 5 Department of Pathology and Laboratory Medicine, Perelman School of Medicine, University of Pennsylvania, Philadelphia, PA 19104 USA; 6 Present Address: Viela Bio, Gaithersburg, MD 20878 USA; 7 Present Address: Bristol-Myers Squibb, Princeton, NJ 08543 USA

## Abstract

Although the aetiology of systemic lupus erythematosus (SLE) is unclear, dysregulated B cell responses have been implicated. Here we show that an unusual CD11c^hi^T-bet^+^ B cell subset, with a unique expression profile including chemokine receptors consistent with migration to target tissues, is expanded in SLE patients, present in nephrotic kidney, enriched for autoreactive specificities and correlates with defined clinical manifestations. IL-21 can potently induce CD11c^hi^T-bet^+^ B cells and promote the differentiation of these cells into Ig-secreting autoreactive plasma cells. While murine studies have identified a role for T-bet-expressing B cells in autoimmunity, this study describes and exemplifies the importance of CD11c^hi^T-bet^+^ B cells in human SLE.

## Abstract (web summary)

Systemic lupus erythematosus (SLE) is associated with altered B cell responses but the underlying aetiology is still unclear. Here the authors show that a CD11c^hi^T-bet^+^ B cell subset with a unique phenotype and transcriptome is increased in patients with SLE, can be expanded by IL-21, and may contribute to autoimmune responses in SLE.

## Introduction

Systemic lupus erythematosus (SLE) is an autoimmune disease of which the aetiology is unclear, although dysregulation of B cell function is believed to have a key role in disease pathogenesis^1^. There are several reports of altered B cell phenotypes in individuals with SLE. Conventionally, CD27 is used as a marker of memory B cells, where CD27/CD70 interactions are involved in the regulation of B cell activation and plasma cell differentiation^2,3^. However, a growing body of literature suggests that subsets of CD27^−^ memory B cells also exist. Memory B cells that lack CD27 expression and co-express CD11c and FcR-like antigen (FcRL4/IRTA-1) are described in the tonsil^4^. In SLE, Sanz and colleagues report a population of autoreactive memory B cells that lacks CD27 expression and is associated with clinical manifestations of lupus^5^. Others also observe CD27^−^ memory-like B cells in SLE that are defined by high spleen tyrosine kinase (SYK) or CD95 expression, that similarly correlate with disease activity^6,7^. Furthermore, other B cell populations are described in SLE including CD19^hi^CXCR3^hi^ B cells that associate with poor clinical outcomes after rituximab treatment^8^, or CD24^−^-activated naive B cells that may be precursors of plasma cells^9^.

Another population of B cells described to be increased in autoimmune disease is a B cell subset that expresses CD11c, a marker traditionally associated with dendritic cells. In rheumatoid arthritis (RA), Sjögren’s Syndrome and common variable immunodeficiency disorder, CD11c^+^ B cells are expanded^10–12^. Moreover CD11c^+^IgD^−^CD27^−^ 'double-negative' B cells in multiple sclerosis are reported to be present in both the peripheral blood as well as the cerebrospinal fluid^13^. B cells that express CD11c are also observed in parasitic disease, after malarial infection^14^. In mice, and in women with RA, CD11c^+^ B cells accumulate with age, thus termed age-associated B cells (ABC)^11,15^. These ABCs express the T-box transcription factor (T-bet), and require both Toll-like receptor (TLR) signalling and T-bet for their generation^11,15–17^. Furthermore, the complementarity-determining regions of murine ABCs contain a substantial number of somatic hypermutations and require both CD40L and MHC class II for their development, suggesting that interactions with activated T cells in the germinal centre may be required for their generation^18^.

In murine B cells, T-bet expression drives class switch recombination (CSR) to IgG2a^11,19,20^, while inhibiting CSR to IgG1 and IgE, resulting in protection from allergic inflammation^21^. In vivo, T-bet is critical for maintaining antigen specific memory of IgG2a B cells^20^. In autoimmunity, loss of T-bet in murine models of lupus results in greatly reduced B cell-driven disease manifestations^19,22^. Furthermore, removal of CD11c^+^ B cells from mice immunised with TLR7 agonist markedly reduces anti-Smith (Sm) titres^11^. Taken together, these studies suggest that in pre-clinical murine models, the majority of B cells with autoreactive specificity originate from CD11c^+^T-bet^+^ B cells. Additionally, in the context of influenza immunisation, CD21^lo^ B cells with a phenotype similar to ABCs are described to be post-germinal centre memory B cells^23^.

In humans, T-bet expression can be induced in B cells by IL-27, IFNγ^24^ or IL-21^16,25,26^ and can be expressed by CD11c^+^ B cells of healthy individuals^27^. In both humans and mice, B cell receptor (BCR)/TLR9 co-engagement results in cell cycle arrest and subsequent cell death of B cells; when rescued from this TLR9-dependent checkpoint by co-stimulation with anti-CD40 and IL-21, B cells adopt the T-bet^+^ cell fate^28^. However, neither T-bet expression in B cells from SLE patients, nor the potential contribution of these cells to disease manifestations has been systematically investigated.

Clearly B cells are dysregulated in lupus. One of the most potent cytokines that regulates B cell function is IL-21^29,30^. IL-21 belongs to the common γ receptor family of cytokines and plays a non-redundant role in driving plasma cell differentiation^31–35^. Depending on the signals that B cells receive, IL-21 can induce a range of responses including B cell activation, proliferation, differentiation or death^29^. IL-21 can also influence the ability of B cells to act as suppressor cells either by directly upregulating granzyme B^36^, or by driving differentiation of IL-10-producing regulatory B cells^37^. Activation of B cells from healthy donors with IL-21 co-stimulation can upregulate SYK expression associated with hyporesponsiveness of BCR signalling^38^. Single-nucleotide polymorphisms in both IL-21 and the IL-21 receptor (IL-21R) have also been associated with susceptibility to SLE^39,40^. Moreover, both soluble IL-21 and IL-21 producing T cells are elevated in blood of lupus patients^41–44^, where IL-21^+^ T cells correlate with frequency of memory B cells^43^ and thus, overexpression of IL-21 may contribute to clinical manifestations of disease through propagation of pathogenic autoantibodies.

In this study, we examine a large cohort of SLE patients in order to further define altered B cell subpopulations in SLE and possible links to IL-21. Here, we describe a population of CD11c^hi^ B cells that are poised to differentiate into Ig-producing plasma cells with autoreactive specificities. These findings may suggest that CD11c^hi^ B cells contribute to the pathogenesis of SLE through the generation of autoreactive plasma cells and indicate that targeting CD11c^hi^ B cells may have therapeutic benefit.

## Results

### CD11c^hi^ B cells correlate to disease manifestations in SLE

**Fig. 1:**
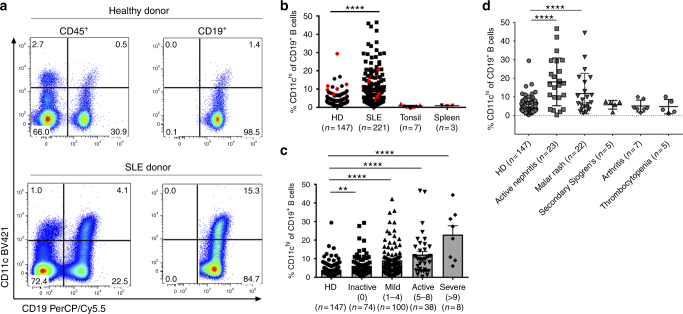
CD11c^hi^ B cells are expanded in SLE and correlate to SLEDAI and specific clinical manifestations. **a**–**d** % CD11c^hi^ B cells (of CD19^+^ cells) was determined as shown in **a**. **b** Distribution of %CD11c^hi^ B cells (of CD19^+^ cells) (healthy donors *n* = 147 (123 unique donors, 24 of which are repeats) or SLE patients *n* = 221 (112 unique donors, 109 of which are repeats), tonsil or spleen. Male donors labelled in red. **c** %CD11c^hi^ B cells (of CD19^+^cells) isolated from blood of healthy donors or SLE patients grouped by SLEDAI range. **d** %CD11c^hi^ B cells (of CD19^+^cells) from healthy donors was compared to SLE with different clinical manifestations. Patients with more than one manifestation were excluded (*n* = 10). **b**–**d** (Data are represented as mean ± SEM, non-parametric Mann–Whitney test, **p < 0.01, ****p < 0.0001)

B cell phenotype was evaluated by flow cytometry from blood samples of over 200 SLE patients and 147 matching healthy donors (Supplementary Table 1), and compared to disease severity scores and other measures of disease activity. Strikingly, a high proportion of circulating B cells isolated from SLE patients displayed greatly increased density of CD11c, comparable to the density observed on dendritic cells isolated from the same individual (Fig. 1a). The frequency of these CD11c^hi^ B cells was increased in SLE patients compared to healthy donors (Fig. 1b). These B cells were present in tonsil and spleen, but at very low frequencies, suggesting that CD11c expression did not simply denote an activated phenotype (Fig. 1b). To evaluate the relationship with disease activity, the percentage of CD11c^hi^ B cells was compared to SLE patients grouped according to the SLEDAI disease activity index. Notably, the frequency of CD11c^hi^ B cells was increased in patient groups with higher SLEDAI and was highest in the most severe patients with SLEDAI of 9 or above (Fig. 1c). We next determined if CD11c^hi^ B cells were preferentially expanded in SLE with distinct clinical manifestations. CD11c^hi^ B cells were found at the highest frequencies in SLE patients with coincident active nephritis and malar rash (Fig. 1d), suggesting that this B cell subset has the potential to associate with specific clinical disease manifestations. Notably, the SLEDAI of patients with these specific disease manifestations was not different from those with other symptoms, such as arthritis, where CD11c^hi^ B cells were not enriched (Fig. 1d, Supplementary Fig. 1a), illustrating the specificity of this clinical association. When patients were stratified based on immunomodulatory drug treatment, such as CellCept (MMF) or cyclophosphamide (CTX), no association was found between MMF and percent CD11c^hi^ B cells, whereas an association was found for CTX (Supplementary Fig. 1b). As the more severe patients were not necessarily on CTX, the correlation we observed between CD11c frequency and SLEDAI is unlikely due to associations with CTX treatment.

Other measures of disease activity were also compared to CD11c^hi^ B cells where a significant negative correlation with C3 and C4 concentrations was found (*r* = −0.38 and −0.32, respectively, *p* < 0.0001, Pearson correlation test), a weak positive correlation with erythrocyte sedimentation rate was obtained (*r* = 0.17, *p* = 0.01, Pearson correlation test), and no association with C-reactive protein noted (Supplementary Fig. 1c).

**Fig. 2:**
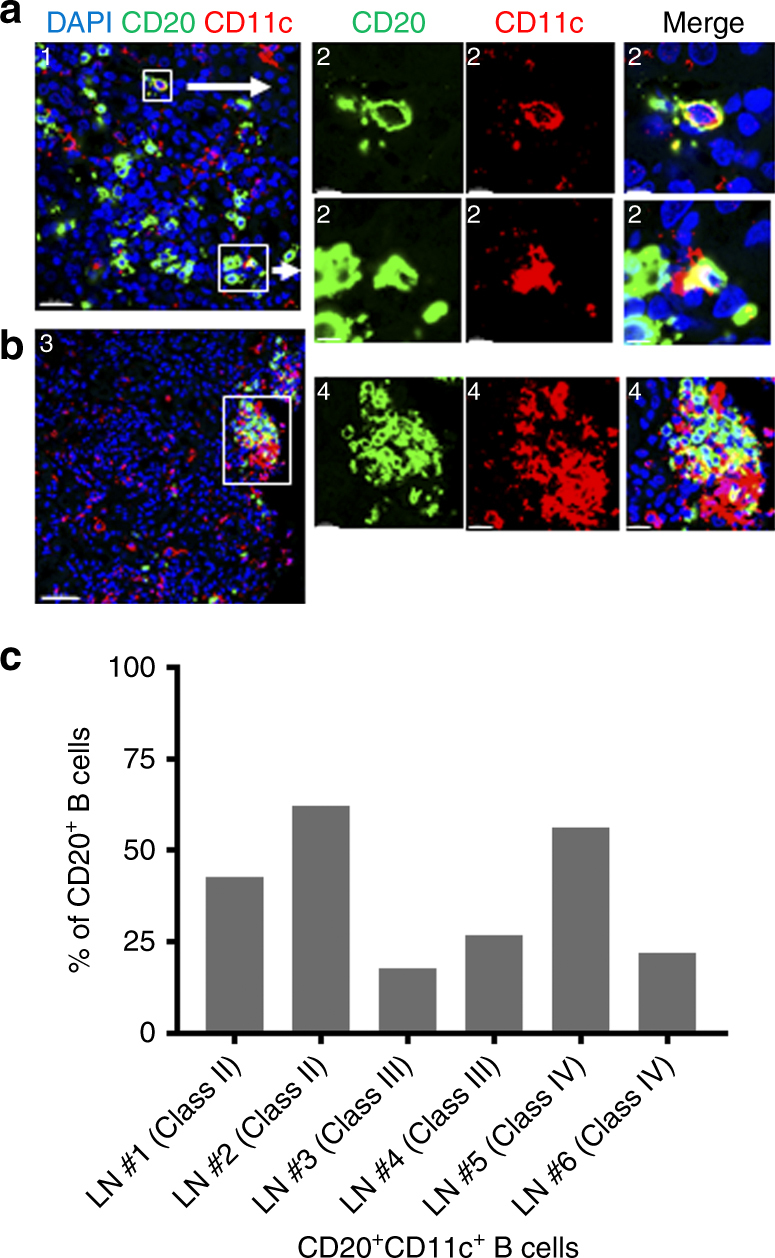
CD11c^hi^ B cells are present in nephritic kidney in SLE. Nephritic kidneys from SLE patients were examined for the presence of CD11c^hi^ B cells. A total of 11 kidney biopsies were examined, of which 6 contained B cell infiltrate as defined in methods where all 6 showed presence of CD20^+^CD11c^+^ double positive B cells. Green shows CD20, red shows CD11c, and yellow indicates double positive cells. **a** Kidney section of lupus nephritis Class II. **b** Kidney section of lupus nephritis Class IV-b. Space bars show length as indicated. Scale bars = 1, 20 μM; 2, 3 μM; 3, 50 μM; 4, 10 μM. **c** Frequencies of CD20^+^CD11c^+^ double positive B cells present in nephritic kidneys as indicated compared to CD20^+^ B cells from six unique lupus nephritis donors

Tubulointerstitial inflammation in lupus nephritis (LN) has been shown to be a predictor of progression to renal failure^45^. As the highest association of CD11c^hi^ B cells was found in lupus patients with active nephritis, we examined if CD11c^hi^ B cells could be found in the target tissues of nephritic kidney. Thus, we examined kidney biopsies of active LN patients for the presence of CD20^+^CD11c^+^ B cells. Notably, CD20^+^CD11c^+^ B cells were noted in all biopsies that contained B cell infiltrate (Fig. 2a–c), either as a diffuse staining pattern (Fig. 2a), or often contained within an ectopic-like follicle (Fig. 2b). These data demonstrate that CD11c^+^ B cells are not only present in the circulation, but have the capacity to migrate into target tissues.

### Phenotypes of CD11c^hi^ B cells from SLE patients

**Fig. 3:**
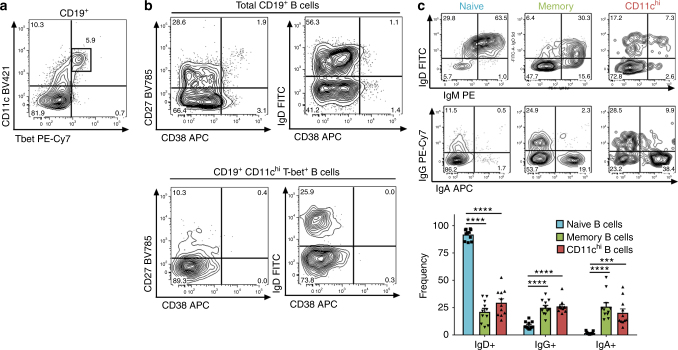
CD11c^hi^ B cells express T-bet and largely do not express CD27 or CD38 and contain switched B cell receptors. **a**, **b** %CD11c^hi^ T-bet^+^ B cells (of CD19^+^cells) in blood was determined in SLE and cell surface phenotype determined. Data representative of 21 unique healthy donors and 39 unique SLE with four repeat SLE donors. **c** Cell surface expression of IgD by IgM or IgG by IgA was determined on CD19^+^CD11c^−^CD27^−^ naive (blue), CD11c^−^CD27^+^ memory (green), or CD11c^hi^ (red) B cells from SLE (Data are represented as mean ± SEM, unpaired *t* test with Welch’s correction, ***p < 0.001, ****p < 0.0001, *n* = 11)

Next, we determined if these CD11c^hi^ B cells from this SLE cohort expressed the transcription factor T-bet as T-bet has been described in B cells from both humans and mice^10,17,21,25,26,46^. As shown in Fig. 3, nearly all CD11c^hi^ B cells present in SLE patients express T-bet (Fig. 3a, Supplementary Fig. 2a). The CD11c^hi^ T-bet^+^ B cells were largely found not to express CD38 and express low levels of the memory B cell antigen CD27, with a portion expressing IgD (Fig. 3b). While a fraction of the CD11c^hi^ B cells expressed IgD, an equal portion expressed IgG or IgA, suggesting that these post-switched CD11c^hi^ B cells are antigen experienced, and may represent post-germinal centre B cells (Fig. 3c). CD11c^hi^ B cells present in healthy individuals also expressed T-bet but were reduced compared to SLE patients (Supplementary Fig. 2b, c).

**Fig. 4:**
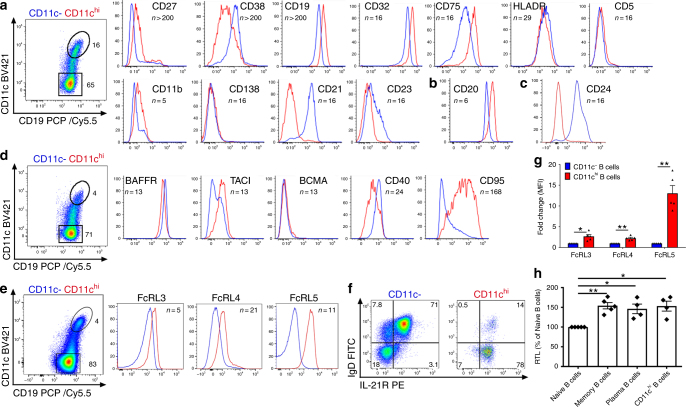
Phenotype of CD11c^hi^ B cells from SLE patients. **a**–**e** Phenotype of CD11c^−^ (blue) or CD11c^hi^ (red) CD19^+^ B cell subsets as indicated was determined in SLE where a unique donor is shown for each panel. **a** Representative phenotype of B cell subsets from the same SLE donor. **b**, **c** CD20 or CD24 expression in B cell subsets from separate SLE donors. **d** Expression of TNF family members was determined on SLE B cell populations as indicated. **e** FcRL family members were assessed on SLE B cell populations as indicated from the same donor. **f** Expression of IgD by IL-21R was determined on SLE B cell populations as indicated. **g** Fold change of FcRL measured as Mean Fluorescence intensity (MFI) of B cell subsets compared to CD11c^−^ B cells as indicated. (Data are represented as mean ± SEM, unpaired *t* test with Welch’s correction *p < 0.05, **p < 0.01, *n* = 5 SLE). **h** Relative telomere length (RTL) was determined in B cell populations as indicated and plotted as % naive B cells. Sorting strategy shown in Supplementary Fig. 11a (Data are represented as mean ± SEM, unpaired *t* test with Welch’s correction, *p < 0.05; **p < 0.01, *n* = 5 unique donors in independent experiments

In order to further characterise CD11c^hi^ B cells, these cells were examined for cell surface expression of both conventional and unconventional B cell antigens. In the majority of B cells examined from over 200 SLE patients, CD11c^hi^ B cells were found to be largely CD27^lo^CD38^lo^ (Fig. 4a). Further characterisation of these cells revealed that CD11c^hi^ B cells expressed higher density of CD19, CD20, CD32 (FcγR), the sialyltransferase antigen CD75^47^, and similar densities of HLA-DR as compared to the CD11c^−^ B cells (Fig. 4a, b). Although in mice, CD11c^+^ ABC express CD5, CD11b and the plasma cell antigen, syndecan1/CD138^11^, in SLE, these CD11c^hi^ B cells were largely negative or expressed low densities of these antigens, and were largely negative for CD21 and CD23. Expression of CD24 was also evaluated where high density of CD24 is observed on the majority of CD27^+^ memory B cells^48^. While CD11c^−^ B cells, as expected, expressed CD24, the CD11c^hi^ B cells did not appear to express this antigen (Fig. 4c).

Upon further analysis of TNF receptor family members, it was revealed that these CD11c^hi^ B cells expressed a high density of BAFF receptor (BAFFR), intermediate densities of transmembrane activator and CAML interactor (TACI) and minimal B-cell maturation antigen (BCMA) that is expressed by plasma cells (Fig. 4d). Unexpectedly, these B cells expressed a low density of CD40, while expressing high density of the apoptotic antigen Fas/CD95. The phenotype of these CD11c^hi^ B cells was found to be similar to that of ABC’s described previously in mice that express CD11c, and are CD21^−^CD23^−^CD95^+^ and largely IgD^lo^BAFFR^+^,TACI^+^ and BCMA^[−15^.

A population of CD27^−^CD11c^+^ B cells has been described in the tonsil that express FcRL4^4^, or in the peripheral blood of healthy individuals that express FcRL5^27^. As shown in Fig. 4e, blood CD11c^hi^ B cells expressed low densities of FcRL3 and FcRL4, although at higher levels than the CD11c^−^ B cells (Fig. 4e, g). Strikingly, however, these cells were found to uniformly express very high densities of FcRL5 (Fig. 4e, g). IL-21R is expressed at high densities on naive B cells, but the expression is downregulated on CD27^+^IgD^−^ or IgD^+^ memory B cells^49^ (Fig. 4f). Notably, similar to naive B cells, CD11c^hi^IgD^−^ and CD11c^hi^IgD^+^ B cells were found to express high densities of IL-21R (Fig. 4f).

In order to understand how CD11c^hi^ B cells from healthy donors (albeit lower in number) compared to those present in SLE, cell surface phenotype was examined. As shown in Supplementary Fig. 3 expression patterns of all antigens examined by flow cytometry was similar between healthy donors and SLE patients, suggesting similarities of these CD11c^hi^ B cells between healthy and autoimmune individuals. It is important to point out that although CD27 is ~10 fold increased in CD11c^hi^ compared to CD11c^−^ B cells, CD27 expression of memory B cells is 223.7-fold increased (healthy donors) and 168.5-fold increased (SLE) compared to naive B cells (Supplementary Fig. 3b). Thus, although CD11c^hi^ B cells do express greater CD27 then CD11c^−^ B cells, it is greatly downregulated compared to memory B cells.

To more fully understand the differentiation stage of these B cells, we examined the telomere length of CD11c^hi^ B cells and compared this to the telomere length of other B cell populations from the same donor. Previously, it has been reported that CD27^+^ memory B cells have a longer telomere length than naive B cells, which is believed to be important in the generation of long-lived memory^50^. As shown in Fig. 4h, the relative telomere length (RTL) of CD27^−^CD38^+^ naive B cells was found to be shorter than that of CD27^+^ memory B cells or plasma cells. Notably, the RTL of CD27^−^CD11c^hi^ B cells was comparable to memory B cells and plasma cells, in contrast to naive B cells (Fig. 4h). Taken together, these data suggest that these CD11c^hi^ B cells represent a subpopulation of antigen experienced B cells, despite low expression of CD24, CD27 and high expression of IL-21R.

**Fig. 5:**
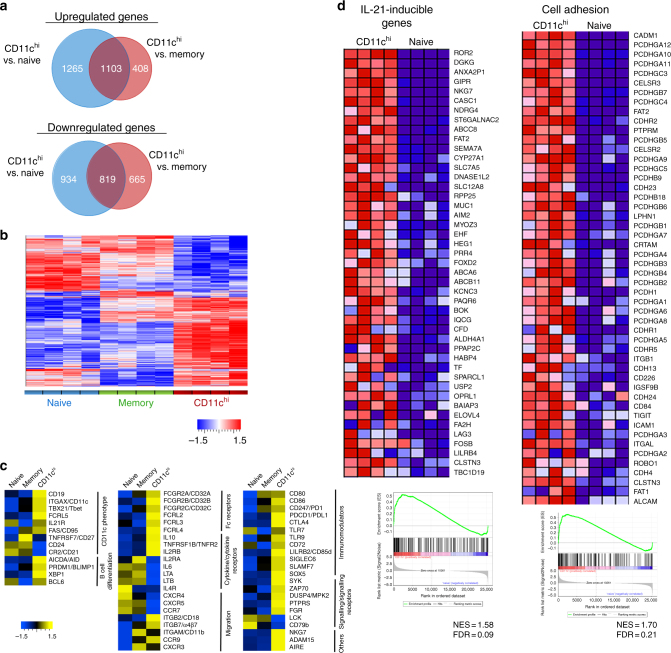
Unique transcriptome profile of CD11c^hi^ B cells in SLE. **a** Venn diagram showing number of genes with significant upregulation or downregulation comparing CD11c^hi^ cells with naive or memory B cells. Sorting strategy shown in Supplementary Fig. 11b. Significance is defined as Fold change (FC) >2 and False discovery rate (FDR) <0.05. **b** Heatmap showing expression pattern of genes with significant change (FC >2, FDR <0.05) in at least one of the group comparisons (CD11c^hi^ vs. naive or CD11c^hi^ vs. memory B cells), and passed cut-off of median counts per million (CPM) >50 in at least one of the three cell populations. Log2 transformed CPM values were used for plotting. Genes were clustered using hierarchical clustering. Red indicates higher expression, and blue indicates lower expression. Colour bar indicates *Z* score. **c** Heatmap showing expression pattern of representative genes with relevant functions. Median log2 transformed CPM values for each cell population were used for plotting. Yellow indicates higher expression, and blue indicates lower expression. Colour bar indicates *Z* score. **d** Gene set enrichment analysis (GSEA) showing pathways enriched in CD11c^hi^ B cells compared to naive B cells. Heatmap shows the expression of the core genes that contribute to pathway enrichment (red, high expression; blue, low expression). NES: normalised enrichment score, FDR: false discovery rate

To better characterise how these cells relate to naive vs. memory B cells, gene expression profiling was performed using RNA sequencing (RNAseq). CD19^+^ B cells from SLE patients were sorted into naive (CD11c^−^IgD^+^CD27^−^), memory (CD11c^−^IgD^−^CD27^+^), pre-switched CD11c^hi^ (CD11c^hi^IgD^+^), or post-switched CD11c^hi^ (CD11c^hi^IgD^−^) B cells. Genes that showed significant up or downregulation were evaluated. Strikingly, CD11c^hi^ B cells showed very different gene expression pattern compared to both naive and memory B cells (Fig. 5a, b). Overall, 1103 genes were significantly upregulated and 819 genes were significantly downregulated in CD11c^hi^ B cells compared to naive and memory B cells, respectively [fold change (FC) >2, false discovery rate (FDR) <0.05; Fig. 5a]. For highly expressed genes with counts per million (CPM) of 50 or above, 256 and 153 genes were significantly up or downregulated, in CD11c^hi^ B cells compared to both naive and memory B cells. (Supplementary Fig. 4a). IgD^+^CD11c^hi^ were remarkably similar to IgD^−^CD11c^hi^ B cells (Supplementary Fig. 4a, b). Venn diagram shows that for the highly expressed genes of over 50 CPM only 17 and 61 genes were up, or downregulated, respectively, comparing IgD^−^CD11^hi^ to IgD^+^CD11^hi^ B cells.

### CD11c^hi^ B cells transcriptome from SLE and RA patients

Further inspection of the genes that were differentially expressed showed that the key phenotypic surface markers of CD11c^hi^ B cells are corroborated by mRNA expression such as *CD19*, *MS4A1* (*CD20*), *ITGAX* (*CD11c*), *TBX21* (*T-bet*), *CR2* (*CD21*), *FCER2* (*CD23*), *FCGR2B* (*CD32B*), *CD24*, *CD27*, *CD38*, *CD40* and *IL-21R* (Fig. 5c, Supplementary Note 1). Notably, RNAseq revealed that CD11c^hi^ B cells appeared related to plasma blasts with upregulation of *PRDM1* (*BLIMP1*), *AICDA* (*AID*), and *XBP1*, as well as genes upregulated in SLE plasma cells^51^, such as *BMP6*, *EMP3* and *S100A4* (Supplementary Note 1). As CD11c^hi^ B cells express low densities of CD27, CD38, and CD138, this suggests that these cells may be precursors of plasmablasts. Presence of CD11c^hi^ B cells in the tissue led us to examine chemokine receptors to better understand their migration capabilities. Our data show that inflammatory chemokine receptors, *CXCR3* and *CCR9*, used by plasmablasts and IgA^+^ antibody secreting cells to enter sites of inflammation and mucosa respectively^52^, were upregulated by CD11c^hi^ B cells compared to naive B cells. Moreover, homoeostatic chemokine receptors necessary for migration into lymphoid organ (*CXCR4*, *CXCR5* and *CCR7*) were downregulated on CD11c^hi^ B cells, although abundantly expressed by naive B cells^52^ (Fig. 5c, Supplementary Note 1). Additional genes and pathways associated with CD11c^hi^ B cells include: BCR signalling (increased *SYK* and *CD79B*), immunomodulation and activation (increased *TLR9*, *CD80*, *CD86* and *CD72*, capable of downregulating CD27^53^), Fc receptor family members involved in Ig and immune complex binding (increased *FcRL2/3/4/5* and *CD32A/B/C*), and immune inhibitors (increased *PD1*, *PDL1*, and *CTLA4*). Furthermore, our data show that cytokine and cytokine receptors are uniquely expressed in these B cells, including gamma-c family receptors (low *IL4R*, *IL2RA* and high *IL2RB*, *IL21R*), and TNF family members (low *LTA*, *LTB*, and high *TNFRSF1B* (*TNFR2*), *TNFSF14* (*LIGHT*), *TNFRSF19* (*TROY*)), as well as unusual expression of cytokines involved in plasma cell differentiation and survival (low *IL6* and high *IL10*) (Fig. 5c and Supplementary Note 1). Lastly, CD11c^hi^ B cells express minimal levels of *CD5* or *CD138*, consistent with cell surface phenotype, but do express higher level of *CD11b*, compared to naive B cells, as described in murine ABCs^11^ (Supplementary Note 1).

To assess the biological processes and signalling pathways of CD11c^hi^ B cells, gene set enrichment analysis (GSEA) was performed. Importantly, pathway enrichment showed that IL-21-inducible genes were found to be upregulated in CD11c^hi^ B cells compared to naive B cells, suggesting these cells were activated by IL-21 in vivo, as well as genes associated with cell adhesion (Fig. 5d).

CD11c^+^CD27^hi^CD5^+^ “ABCs” have been reported in RA^11^, which appear to express a distinct B cell phenotype to that which we described here. In order to address if cells of similar phenotype to the CD11c^hi^ B cells present in SLE are also found in patients with RA, we examined blood B cells from RA patients. As shown in Supplementary Fig. 4c–d, an increase of CD11c^hi^ B cells was noted in RA patients compared to healthy controls, whose demographics are shown (Supplementary Table 2). The CD11c^hi^ B cells in this RA cohort was not found to associate with age (Supplementary Fig. 4e), contrary to previous reports^11^. Further studies of RNAseq analysis revealed that these CD11c^hi^ B cells from RA patients share mRNA expression profiles similar to that found in CD11c^hi^ B cells of SLE, including altered expression of *CD19*, *CD11c*, *T-bet*, *FcRL5*, *CD32B*, *CD21*, *CD23*, *CD24*, *CD27*, *CD38* and *CD40* compared to other B cell subsets (Supplementary Fig. 4f, g). Of interest, CD11c^hi^ B cells sorted from both SLE and RA patients also shared unique transcriptome profile in regard to mRNA expression of cytokine/cytokine receptors, Fc receptors, migration molecules, transcription factors, signalling factors and other pathways (Supplementary Note 2). RNAseq analysis of CD11c^hi^ B cells from healthy donors revealed that while some key genes such as *ITGAX*, *TBX21*, *CD24*, *CD38*, *FCRLs* are regulated comparable to RA and SLE patients, the majority of genes did not share similar transcriptome pattern (Supplementary Note 3). Taken together, these data suggest that although CD11c^hi^ B cells of healthy donors and SLE patients share commonalities in surface antigen expression the unique transcriptome patterns may suggest that CD11c^hi^ B cells from SLE have distinct functionalities.

### Links of CD11c^hi^ B cells with plasma cells and autoantibody

**Fig. 6:**
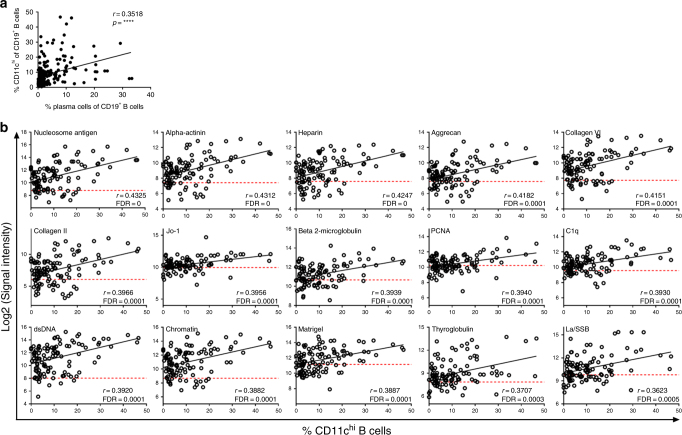
Increase of CD11c^hi^ B cells significantly correlates to plasma cells and a distinct set of IgG autoantibodies in SLE. **a** %CD11c^hi^ B cells (of CD19^+^ cells) was correlated to %CD38^hi^CD27^hi^ plasma cells (of CD19^+^ cells) in SLE blood. (*r*: Pearson correlation coefficient, ****p < 0.0001, *n* = 189). **b** Serum autoantibodies were screened for reactivity to 95 self-antigens. Scatter plots show the top 15 correlated autoantibodies (*r*: Pearson correlation coefficient, FDR: false discovery rate, *n* = 114 total SLE patients with 71 unique donors). Red dashed line indicates median log2 (signal intensity) of the autoantibodies of healthy donors (*n* = 49)

We often noted that SLE patients with a high frequency of CD11c^hi^ B cells also displayed large percentage of CD19^+^CD38^hi^CD27^hi^ plasma cells. Correlations of CD11c^hi^ B cells to frequency of plasma cells present in SLE blood showed an association of these two B cell subsets (Fig. 6a). Thus, we next addressed whether the frequencies of CD11c^hi^ B cells were also associated with lupus-associated autoantibodies. The autoantibody profile of this cohort of lupus patients was examined by assessing reactivity against a panel of 95 autoantigens and compared to frequencies of CD11c^hi^ B cells present in the blood as well as compared to the level of reactivity from sera of healthy donors. Out of all the autoantigens tested, a distinct subset of over half (55) of the autoantibodies showed a significant correlation (Pearson correlation test, FDR ≤0.05) to CD11c^hi^ B cells (compare Supplementary Table 3 to Table 4). Levels of several anti-nuclear autoantibodies associated with SLE^54^ correlated with CD11c^hi^ B cells, including antibodies to dsDNA, nucleosome, several histones, RNP, Smith, La, and chromatin (Fig. 6b, Supplementary Table 3). Additional autoantibodies that correlated with these cells, including antibodies to alpha-actinin, C1q, collagen X, histone H1 and aggrecan, have been reported to be present in LN^55,56^ (Supplementary Table 3). Importantly, the majority of the SLE-associated autoantibodies fell above the levels noted in healthy donors. Further, no significant correlation (as defined by Pearson correlation test *p*-value <0.05) of CD11c^hi^ B cell frequency was found to any of the 95 autoantibodies in healthy donors (Fig. 6b, Supplementary Fig. 5). Taken together, our data suggest a link between CD11c^hi^ B cells with blood plasma cells and a defined set of autoantibody specificities in SLE. However, these data do not rule out other potential B cell populations in SLE patients that could show similar correlations.

### CD11c^hi^ B cells are poised to become plasma cells

**Fig. 7:**
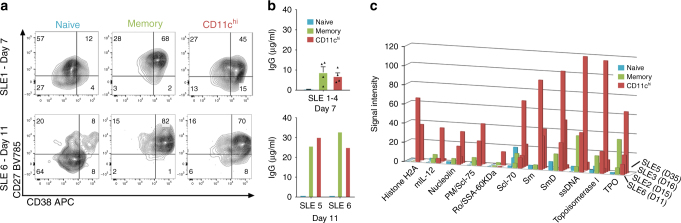
CD11c^hi^ B cells are poised to differentiate into plasma cells and are the major producers of autoantibody. **a**–**c** B cells from SLE patients were sorted into CD19^+^CD11c^−^CD27^−^ naive (blue), CD11c^−^CD27^+^ memory (green), or CD11c^hi^ (red) B cells and added to anti-CD3-activated T cells. Sorting strategy shown in Supplementary Fig. 11c. For several experiments, an early and late time point was evaluated from separate culture wells. Each SLE number represent an independent donor examined at one or more time points. **a** After 7 or 11 days of culture, CD19^+^CD3^−^ B cells were analysed for CD27^+^CD38^hi^ plasma cell phenotype. **b** IgG in the supernatant was determined by ELISA (for day 7, data are represented as mean ± SEM of four independent experiments, or day 11, two independent experiments). **c** Autoantibodies in the supernatant were screened for reactivity to 95 self-antigens on culture day indicated. Bar plot shows significantly increased autoantibodies (*p* < 0.05) in CD11c^hi^ vs. naive B cells as described in Methods

Based on the positive association of CD11c^hi^ B cells with plasma cells and serum autoantibodies, we next asked if these B cells were poised to differentiate into plasma cells, or were anergic as described of B cells with a similar phenotype in RA^10^. Previously, we have shown in vitro, that activated T cells induce plasma cell differentiation of total human blood B cells^34^. Thus, CD19^+^ B cells from SLE patients were sorted into CD11c^−^CD27^−^ naive, CD11c^−^CD27^+^ memory, or CD11c^hi^ B cells and cultured with activated T cells. As shown in Fig. 7a, activated T cells were much more effective at inducing plasma cell differentiation (as defined by CD19^+^CD27^hi^CD38^hi^ cells) from CD11c^−^CD27^+^ memory B cells compared to CD11c^−^CD27^−^ naive B cells. Notably, CD11c^hi^ B cells responded efficiently to the activated T cells, with 45 and 70% of B cells displaying a plasma cell phenotype after day 7 or 11 days of culture, respectively (Fig. 7a). Consistent with this, sorted naive, memory and CD11c^hi^ B cells were found to produce IgG after activation with T cells, where total IgG levels increased with length of culture (Fig. 7b). Similar to plasma cell phenotype, IgG levels from cultures of CD11c^hi^ B cells with activated T cells was similar to memory, rather than naive B cells. Importantly, unlike plasma cells, these CD11c^hi^ B cells freshly isolated from SLE donors did not spontaneously produce IgG as determined by EliSpot, nor in culture in the absence of activated T cells (Supplementary Fig. 6).

The next question we addressed was whether these antibodies contained autoreactive specificities. To this end, supernatant from the above co-cultures were tested for reactivity against a panel of 95 autoantigens. Strikingly, plasma cells that were differentiated from the CD11c^hi^ B cells produced significantly higher levels of autoantibodies compared to those differentiated from naive or memory B cells. In the cohort of SLE patients examined, out of the 95 specificities tested, 11 autoantibodies were found to be significantly increased (*p*-value <0.05, as determined by Linear model based group comparison as described in Methods) in the CD11c^hi^ B cell group, compared to those from the naive B cell group (Fig. 7c). Moreover, seven autoantibody specificities were found to be significantly increased (*p*-value <0.05, as determined by Linear model-based group comparison as described in Methods) in the CD11c^hi^ B cell group compared to the memory B cell group (Supplementary Fig. 7). Importantly, no autoantigen specificities were significantly increased in the memory B cell group compared to CD11c^hi^ B cells. The increased level of autoantibodies noted in the CD11c^hi^ B cell group did not appear to be simply due to augmented production of total IgG, as both memory and CD11c^hi^ B cells were found to secrete similar amounts of total IgG on day 7 or day 11 of culture (Fig. 7b).

We also addressed if CD11c^hi^ B cells isolated from healthy donors had the capacity to differentiate into plasma cells. As shown in Supplementary Fig. 8, both purified memory and CD11c^hi^ B cells efficiently differentiated into plasma cells and produced IgG upon co-culture with activated T cells (Supplementary Fig. 8a, b, Supplementary Table 5). Previously, we reported that plasma cell differentiation of total human B cells by activated T cells require de novo production of IL-21^34^. Consistent with our previous data, plasma cell differentiation of both memory and CD11c^hi^ B cells was largely inhibited upon neutralisation of either IL-21 or CD40L (Supplementary Fig. 8c).

### IL-21 regulates expansion of CD11c^hi^ B cells

**Fig. 8:**
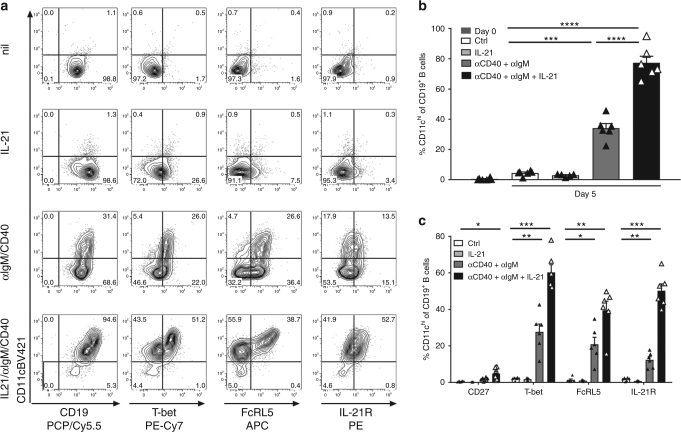
IL-21 co-stimulates the CD11c^hi^ B cell phenotype. CD11c^−^ naive B cells were sorted from the blood of 6 unique SLE patients and activated with a combination of stimulators as indicated. **a**–**c** Cell surface phenotype was evaluated after 5 days of culture of CD19^+^ B cells excluding IgD^−^CD38^hi^ plasma cells. **b** Enumeration (mean ± SEM) of the frequencies of CD11c^hi^ cells of CD19^+^ B cells either post sort (day 0, *n* = 6) or after 5 days of culture. **c** Enumeration (mean ± SEM) of the frequencies of CD11c^hi^CD19^+^ B cells with the indicated phenotype. **b**, **c** *n* = 6 independent experiments of unique SLE donors, expect for IL-21 stimulation only, where *n* = 5, *p < 0.05, **p < 0.01, ***p < 0.001, ****p < 0.0001 as determine by unpaired *t* test with Welch’s correction

Next, we investigated whether IL-21 was involved in the differentiation of these cells, as unlike other memory B cell populations, CD11c^hi^ B cells expressed high density of IL-21R. To this end, CD11c^−^ naive B cells were isolated from the peripheral blood of SLE patients and stimulated with a combination of activators. Remarkably, IL-21 in combination with anti-IgM and anti-CD40 resulted in upregulation of CD11c expression in the majority of naive B cells (Fig. 8a, Supplementary Table 6). While anti-IgM with anti-CD40 in the absence of IL-21 increased CD11c levels in approximately a third of the naive B cells, IL-21 was required for maximum expression (Fig. 8a, b).

We next asked if IL-21 co-stimulation resulted in upregulation of only CD11c, or if IL-21 was driving the differentiation of these cells. Thus, we examined expression of other phenotypic markers. Strikingly, this stimulation cocktail resulted in the differentiation of cells that closely resembled that of CD11c^hi^ B cells present in the blood of SLE patients, namely, low CD27 expression with high densities of T-bet, FcRL5 and IL-21R (Fig. 8a, c). The ability of IL-21 to increase CD11c expression was not unique to SLE naive B cells, as naive B cells from healthy donors also increased CD11c expression after IL-21 co-stimulation (Supplementary Fig. 9a, b). Stimulation through TLR9 did not result in a similar profile but did allow for further B cell expansion.

### CD11c^hi^ B cells in blood do not associate with age

A population of CD11c^+^CD27^+^ B cells has been described to accumulate in the blood of aged women with RA^11^. Analysis of the circulating CD11c^hi^ B cells from healthy donors and this lupus cohort revealed that there was no apparent correlation between the frequencies of CD11c^hi^ B cells to the age of female healthy donors or SLE patients (Supplementary Fig. 10a, b). In fact, the female lupus patients with the highest percent of CD11c^hi^ B cells were present in the younger individuals that tended to decrease with age (Supplementary Fig. 10b). Furthermore, these B cells were also noted in men with SLE where a negative correlation was noted between CD11c^hi^ B cells and age (Supplementary Fig. 10b). Consistent with our studies, B cells of similar phenotype to CD11c^hi^ B cells were also found not to be age associated in healthy donors^57^ or patients with multiple sclerosis^13^.

## Discussion

Here we describe a population of CD11c^hi^ B cells that are highly expanded in lupus and present in target tissues. Our data suggest that CD11c and other integrin and chemokine receptors are guiding these cells to sites of inflammation where they may contribute to immunopathology upon encountering activated T cells and differentiate locally into plasma cells with autoreactive specificities. Further, we show that CD11c^hi^ B cells are associated with LN and malar rash but not with other SLE subtypes. Clinically, the pathophysiology that drives the various SLE sub-manifestations is not well understood. However, both active LN and malar rash are associated with high dsDNA autoantibodies^58^. Moreover, active LN is also associated with hypocomplementemia. Importantly, we show that CD11c^hi^ B cells significantly correlated with both anti-dsDNA levels, as well as with low complement present in the serum. We also show that CD11c^hi^ B cells were poised to differentiate into plasma cells and appear to produce the majority of autoantibodies (including specificities capable of binding nuclear antigens) when compared to plasma cells differentiated from other B cell subsets from the same individual. Our data suggest that CD11c^hi^ B cells may contribute to immunopathology of kidney and skin through local production of autoantibodies with pathogenic potential.

Of note, plasma cells differentiated from CD11c^hi^ B cells also produced significantly higher levels of autoantibody to the RNA-associated autoantigens Sm and SmD, compared to plasma cells differentiated from either naive or memory B cells. Others have described similar findings with B cells that share phenotypic similarities of CD11c^hi^ B cells including CD19^hi^ B cells from SLE donors which can be driven to plasma cells that produce anti-Sm autoantibodies^8^. Autoantibodies present in sera of SLE, including anti-Sm, significantly associate with the frequency of CD19^hi^ or CD27^−^IgD^−^ 'double-negative' B cells^5,8^. The concept that CD11c^hi^ T-bet^+^ B cells in human autoimmune disease are skewed to autoreactive specificities is supported by mouse studies. In mice, CD11c^+^ ABCs stimulated with TLR7 agonist produce anti-Sm autoantibodies where in vivo depletion of these CD11c^+^ ABC, results in substantial reduction of anti-Sm autoantibodies after immunisation with TLR7 agonist^11^. Furthermore, in murine models of lupus, loss of T-bet expression greatly reduces B cell-related disease manifestations^22^, including autoantibody titres, while T cell manifestations are spared^19^, suggesting T-bet expression in B cells is playing a critical role in the regulation of autoreactive responses.

CD11c^hi^ B cells express a unique cell surface and transcriptome phenotype not noted on other B cell subsets. While intermediate densities of CD11c are found on B cells that appear to contain classical CD27^+^ memory B cells, T-bet expression was limited only to those B cells that express very high densities of CD11c, comparable to that noted on dendritic cells. In mice, B cell-expressed T-bet has been shown to be required to drive class switch to IgG2a^19^ and involved with B cell survival^20^. In humans, B cell-expressed T-bet has been described in autoimmunity (this report and refs. ^10,59^), or after parasitic infection^46^, suggesting that this cell population is expanded after chronic B cell activation. As CD11c^hi^ B cells associate with disease severity scores in SLE, this may suggest that increases of these cells in the periphery may also reflect tissue mobilisation by ongoing activation. This is supported by the presence of CD20^+^CD11c^+^ B cells in nephritic kidney, as well as the pattern of expression of chemokine receptors where CD11c^hi^ B cells downregulate lymphoid tissue–chemokine receptors, and upregulate chemokine receptors associated with recruitment into inflamed tissues^52^. These data have clinical implications for patients with LN where we revealed a significant correlation of CD11c^hi^ B cells in these patients. Further studies focusing specifically on this subset will help better define the pathophysiology of LN. CD11c^hi^ B cells may have important implications for identifying SLE patients at risk of developing LN and could prove clinically useful as a predictive biomarker, which is largely not available for LN.

Another unique marker of these cells is FcRL5 where high expression of this antigen was limited to CD11c^hi^ B cells. FcRL is a family of proteins that share homology with the Fc gamma receptor I, where FcRL5 binds IgG of all subclasses, with strongest binding by IgG1 and IgG2^60^. FcRL5 signalling can result in both inhibition as well as stimulation, when B cells are co-stimulated through either the BCR or TLR9, respectively^61,62^. Such a dual response is possible due to the association of FcRL5 with both activating (ITAM) and inhibitory (ITIM) motif elements^63^. Other FcR, or FcR-like receptors that express ITAM and ITIM motifs were also upregulated in these CD11c^hi^ B cells. This includes high expression of CD32B (ITIM), unusual expression of CD32A (ITAM) that is not expressed on other B cells subsets, and CD32C, whose extracellular domain encompasses the CD32B antigen, with the intracellular region containing the CD32A ITAM motif^64^, and LILRB2/CD85d, an Ig-like receptor that contains an ITIM domain. This suggest that these FcR or FcR-like receptors compete for binding of Ig immune-complexes present in SLE, that could result in either a positive or negative signal dependent on complex interactions of these molecules.

IL-21, a master regulator of B cell responsiveness, has wide reaching actions in determining how B cells respond to their environment, capable of inducing B cell activation, CSR, plasma cell differentiation or death, depending on the context of co-stimulation^29^. Normally IL-21R is down regulated when a B cell differentiates from a naive to a memory B cell^49^. These CD11c^hi^ B cells, that appear antigen experienced, uncommonly express high densities of IL-21R, and mRNA associated with IL-21R signalling. In cultures of human naive B cells, IL-21 co-stimulation drove the differentiation of CD11c^+^T-bet^+^FcRL5^+^IL-21R^+^ B cells, consistent with other studies that show IL-21 induced T-bet expression in B cells after TLR stimulation^16,28^. Moreover, IL-21 was involved in the differentiation of these cells to plasma cells, similar to what we have previously reported for total human B cells^34^. Importantly, IL-21, and IL-21-producing T cells are increased in SLE^41,42^ and correlate to specific B and T cell subsets^41,43^. Taken together, these data suggest that among IL-21’s many roles in activation and differentiation of B cells, over expression of this cytokine in SLE may contribute to autoimmune pathology through influencing the expansion of CD11c^+^ T-bet^+^ B cells with autoreactive specificities.

Other unusual phenotypic components of these memory B cells are lower expression of CD27 and CD40 (compared to memory B cells), which suggests that these B cells may not have productive interactions with T cells that express the ligands for these molecules. However, despite low expression of these antigens, the CD11c^hi^ B cells readily differentiated into plasma cells capable of secreting autoreactive IgG in B cell/T cell co-cultures. Thus, these cells do not appear anergic, as has been reported of B cells with similar phenotype described in RA or Sjögren’s Syndrome^10,12^, but rather responded maximally to activated T cells. Others have reported that a single stimulus was not sufficient to induce activation of memory B cells with a similar phenotype to CD11c^hi^ B cells, but rather require several activation signals to induce proliferation and differentiation^57^, which would be delivered by activated T cells in our system.

In human B cells, CD27 is a differentiation antigen induced after activation of naive B cells that continues to upregulate as memory B cells differentiate into plasma cells. Interaction of CD27 with T cell-expressed CD70 regulates B cell activation, plasma cell differentiation and Ig production^2,3,65^. However, CD70 expressed on activated T cells can also downregulate CD27 expression on B cells^65^. In this regard, T cells isolated from lupus patients over-express CD70, which correlates with disease activity^66^. Taken together, these studies suggest that the CD11c^hi^ memory B cells described here may have recently encountered CD70 expressed on activated T cells, resulting in downregulation of CD27.

In summary, we show that a population of CD11c^hi^ T-bet^+^ B cells is significantly expanded in SLE that may have the capacity to differentiate into autoreactive plasma cells upon encounter with activated T cells. Given their unique phenotype, these cells have the potential to be targeted by biologics, which may prove efficacious in autoimmune diseases where autoantibodies are believed to have a pathogenic role.

## Methods

### Cells from healthy donors

Human whole blood was collected after informed written consent from healthy volunteers recruited by the MedImmune Blood Donor program. Healthy control donors consisted of healthy MedImmune or AstraZeneca employees, who were anonymously enroled in the MedImmune Research Specimen Collection Program. Donors with HIV infection, hepatitis B or C virus, Human T-lymphotropic virus, or syphilis were excluded. Informed written consent for blood draws was obtained from the donor. Peripheral blood mononuclear cells (PBMCs) were isolated from CPT tubes (BD Biosciences) after centrifugation. Naive B cells were negatively selected using MACS cell separation kit #130-091-150 (Miltenyi Biotec), which routinely yielded greater than 85% purity. The degree of purity and initial cell phenotype were determined by flow cytometry for all experiments on day 0.

### B cells from autoimmune donors

Lupus and RA blood samples were obtained from the Warren G. Magnuson Clinical Center Blood Bank (Bethesda, MD) as approved by the National Institute of Arthritis and Musculoskeletal and Skin Diseases/National Institute of Health and isolated as described above. The demographics and clinical characteristics of these donors are shown in Supplementary Table 1 (SLE) and Supplementary Table 2 (RA). Naive B cells from SLE were sorted as CD19^+^CD11c^−^CD27^−^ and post sort purity was greater than 90%.

### B cells from spleen and tonsil

Tonsil (four males, 4, 18, 23 and 37 years old and three females, 19, 22 and 30 years old) and spleen (female, 64 years old, male, 80 and 82 years old) tissue was acquired from the National Disease Research Interchange (Philadelphia, PA). Tissues were cut and mashed gently to remove all lymphocytes. Tonsil cells were lysed with ammonium chloride and washed with RPMI 10% FBS. Splenocytes were isolated by ficoll separation and washed with RPMI 10% FBS. B cells from tonsil and spleen were visualised by flow cytometry as described below.

### Culture conditions of purified SLE B cells

Sort purified SLE naive peripheral blood B cells were cultured at a density of 0.5–1 × 10^5^ B cells per well and healthy donor naive peripheral blood B cells were cultured at a density of 1.5 × 10^5^ B cells per well in 96-well round-bottom plates in a final volume of 200 µl complete medium. Culture medium for all experiments was RPMI 1640 (Invitrogen) supplemented with 10% FCS, penicillin-streptomycin (100 units/ml penicillin, 100 mg/ml streptomycin), 2-mercaptoethanol (55 mM), L-glutamine (2 mM), and HEPES (5 mM). At initiation of culture, B cells were stimulated with a combination of IL-21 (40 ng/ml, MedImmune, LLC), anti-CD40 (0.1 µg/ml, goat IgG, R&D Systems), and anti-IgM F(ab’)_2_ (5.0 µg/ml, Jackson ImmunoResearch Laboratories). The concentration of antibodies used in these experiments was determined based on maximal inhibition achieved in extensive titration studies. B cells were cultured for 5 days prior to examination. The healthy donor naive B cells were also stimulated with CpG-B (1 µg/ml, Invivogen). In order to calculate the total number of CD11c^hi^ B cells present after 5 days of culture, the total number of events within the CD11c^hi^ gate that was collected in 70 µl of FACS buffer on the BD LSR II flow cytometer (BD Biosciences) was back calculated to the total volume of 100 µl.

### Flow cytometry

Freshly isolated lymphocytes of blood, spleen, tonsil or cultured B cells were stained in round-bottom plates for 30 min at 4 °C in a total volume of 100 µl. All fluorochrome-conjugated mouse anti-human mAbs and quantity used for flow cytometry are shown in Supplementary Table 7. Mouse anti-human PE-Cy7-anti-IgG (clone G18-145, BD Biosciences) and APC-anti-IgA (clone IS11-8E10, Miltenyi Biotec) were used for IgG and IgA surface staining. For intracellular T-bet staining, cells were surface stained, fixed using eBio fix/perm kit (eBioscience#00-5523), washed and stained in 1 × eBio fix/perm buffer. Cells were analysed on a BD LSR II flow cytometer (BD Biosciences) using FACSDiva software. Mouse IgG1 kappa Isotype Control, PE-Cyanine7 (eBioscience#25-4714-42) was used as isotype control for T-bet staining. All conditions were collected for the same amount of time, thus the number of events displayed is reflective of the relative cell number.

### Autoantigen arrays

For autoantibodies measure in sera: Healthy donor and lupus patient sera were screened for reactivity to a panel of 95 autoantigens (UT-Southwestern (UTSW) Microarray Core Facility, Dallas, TX) as described^67^. Array signal intensities were normalised and log2 transformation was performed to ensure normal distribution. Pearson correlation test was performed with percentage of CD11c^hi^ cells and values of each autoantigen using R. FDR was calculated using the Benjamini–Hochberg procedure. Autoantibodies with significant correlation were defined as FDR ≤0.05. Heatmap was plotted using R.

For autoantibodies measure in cell supernatant: B cells were sorted from total of 13 SLE patients (for a total of six independent experiments; SLE 1-6) into CD19^+^CD11c^−^CD27^−^ “naive” B cells, CD19^+^CD11c^−^CD27^+^ “memory” B cells, or “CD19^+^CD11c^hi^” B cells and cultured as described below (B cell/T cell co-cultures). Some samples were pooled from multiple donors to achieve sufficient cell numbers for culture: sample SLE1, 2, and 5 were pooled from three donors; SLE3 and 4 were from individual donor; SLE6 was pooled from two donors. Supernatant was collected between 7 and 35 days of culture (day 7 for SLE1 and 4, day 11 for SLE 6, day 15 for SLE2, day 16 for SLE 3, day 35 for SLE5 (for SLE5, naive and CD11c only, no memory condition) and screened for reactivity to a panel of 95 autoantigens, using the UTSW platform as described above. Supernatant from sample SLE 5 and 6 were diluted in order to have sufficient volume for autoantigen arrays. For SLE 6, supernatant from all conditions was diluted by 50%; and for SLE 5, supernatant from all conditions was diluted by 25%. Signal intensities were normalised and log2 transformed, and group comparisons were performed using the R Limma package based on linear model and modified *T* test. Significance was defined as *p*-value <0.05.

### Cell sorting and determination of RTL

B cells were enriched from PBMC from either individual healthy donors or two pooled healthy donors (to increase cell yield) with RosetteSep human B cell enrichment cocktail (StemCell Technologies#15064). The enriched B cells were stained as described above with 5 µl anti-CD19 BUV395, 1 µl anti-CD38 APC, 5 µl anti-CD11c BV421 (All from BD Biosciences) and 10 µl anti-CD27 BV785 (BioLegend) per 1 × 10^6^ cells. Using a FACS Aria Fusion (BD Biosciences), CD19^+^CD11c^−^ B cells were sorted as naive (CD27^−^CD38^+^), memory (CD27^+^CD38^−^/CD38^+^), or plasma cells (CD27^hi^CD38^hi^). CD19^+^CD11c^+^ B cells were sorted as CD27^−^CD38^−^ B cells. Post sort purity was greater than 90%; B cells from five independent donors were examined. In one of the sorts insufficient cells were obtained from CD11c^+^ B cells and plasma cells to extract data. The RTL of sorted B cell populations were determined using Telomere PNA Kit/FITC for flow cytometry (Dako #K5327), per manufacturer’s instructions.

### Sorting and culture of B cell/T cell co-cultures

Peripheral blood B cells were enriched with RosetteSep human B cell enrichment cocktail (StemCell Technologies#15064). The enriched B cells were stained as described above. Using a FACS Aria Fusion (BD Biosciences), B cells were sorted as: CD19^+^CD11c^−^CD27^−^ B cells naive B cells, CD19^+^CD11c^−^CD27^+^ B cells memory B cells or CD19^+^CD11c^hi^ B cells. Some experiments used pooled sorted B cells from multiple donors to achieve sufficient cell numbers for culture. T cell/sorted B cells were cultured as previously described^34^. Briefly, CD4^+^ T cells were mitomycin-C treated (30 μg/ml, Sigma Aldrich) for 30 min at 37 °C then washed and rested in complete media at 37 °C for an additional 30 min. 1.0 × 10^5^ mitomycin-C treated CD4^+^ T cells were cultured with 0.2–0.5 × 10^5^ purified B cells (per 96 well) and co-cultured in a final volume of 200 µl. T cells were stimulated with T cell Activation/Expansion kit (Miltenyi Biotec#130-091-441) in a 2:1 T cell to bead ratio. Culture medium for these experiments was RPMI 1640 (Invitrogen) supplemented with 10% FCS, penicillin–streptomycin (100 units/ml penicillin, 100 μg/ml streptomycin), 2-mercaptoethanol (55 μM), L-glutamine (2 mM), and HEPES (5 mM). After 7 or 11 days of culture, cells were stained by flow cytometry as described above for plasma cell phenotype and IgG in the supernatant was determined as described below. Several experiments were set up in duplicate to allow for analysis of secreted autoantibodies at later culture time points.

### Immunoglobulin production

Secreted Ig was quantified by ELISA after stimulation of B cells for the indicated number of days. Ninety six-well flat-bottom plates were coated overnight at 4 °C with either 5 μg/ml of goat anti-human IgM or goat anti-human IgG diluted in PBS. Plates were washed and blocked with 0.2% BSA in PBS. Supernatants were diluted and incubated in plates overnight. Bound Ig was detected with goat anti-human IgG-alkaline phosphatase (0.2 μg/ml, Bethyl Laboratories) diluted in blocking buffer. Plates were developed with SigmaFast p-Nitrophenyl phosphate Tablets (Sigma Aldrich), and specific absorbance was measured at 405 nm using a SpectraMax microplate reader (Molecular Devices).

### ELISpot assay for detection of IgG-secreting plasma cells

ELISpot was performed according to manufactures instructions (Human IgG ELISpotBASIC HRP, Mabtech). Briefly, PVDF ELISpot plates (MAIPS4510, Millipore) were Ethanol treated and coated with capture antibody (MT91/145, Mabtech) at a concentration of 15 µg/ml in PBS and incubated overnight at 4 °C. Total PBMCs or sorted CD11c^hi^ B cells (CD19^+^CD11c^hi^) or plasma cells (CD19^+^CD27^++^CD38^++^) from SLE donors (*n* = 4) were added to wells at indicated concentrations in complete media and incubated for 16 h at 37 °C. The plates were then washed and incubated with detection mAb (MT78/145, Mabtech) at a concentration of 1 µg/ml for 2 h at RT, followed by streptavidin-horseradish peroxidase conjugate for 1 h at RT and developed with precipitating TMB substrate (Mabtech) for 15 min. The images were captured using an Immunospot reader (Cellular Technology Limited).

### RNAseq and data analysis

Peripheral blood B cells from SLE patients (*n* = 4 independent samples) were isolated using human B cell enrichment kit (StemCell Technologies, catalogue #19054) and stained as described above. Using a FACS Aria Fusion (BD Biosciences), B cells from SLE patients were sorted as: CD19^+^CD11c^−^CD27^−^IgD^+^ naive B cells, CD19^+^CD11c^−^ CD27^+^IgD^−^ memory B cells, CD19^+^CD11c^hi^IgD^+^ B cells or CD19^+^CD11c^hi^ IgD^−^ B cells. B cells from healthy donor (*n* = 4 independent samples) or RA patients (*n* = 4 independent samples) were sorted as: CD19^+^CD11c^−^CD38^inte^ IgD^+^ B naive B cells (*n* = 3 used for analysis), CD19^+^CD11c^−^CD38^inte^ IgD^−^ B cells memory B cells, CD19^+^CD11c^−^CD38^−^IgD^−^ memory B cells or CD19^+^CD11c^hi^CD38^−^IgD^−^ B cells. The purity of the sorted populations was routinely >90%.

RNA was isolated using PicoPure RNA isolation kit (Thermo Fisher Scientific, catalogue#: KIT0204). RNAseq was performed at MedImmune Deep Sequencing and Microarray Core. RNA sequencing data (RNASeq) data was generated using the Illumina standard library preparation and sequencing protocols as described^68^. In brief, mRNA-seq libraries were generated using the TruSeq RNA Sample Preparation kit (Illumina, catalogue #RS-122-2001) and sequenced on the HiSeq 2000 platform according to the manufacturer’s recommendations. Paired end FASTQ files of 90 mer sequence reads were generated. For RNASeq data, the average read count per mate was 50 million. Quality of the RNAseq data, such as the overall sequencing score, over-represented reads, kmer presence, was evaluated using the FastQC package^68^.

For sorted SLE B cell subsets, sequencing reads were aligned to human reference genome hg19 using Hisat2 (v2.0.2)^69^. Default parameters were used. Raw counts were generated using HTseq^70^. Data was normalised and CPM values for each gene were generated using the Deseq2 package. Group comparisons were performed in Deseq2 using generalised linear model assuming negative binomial distributions^71^. False discovery rate (FDR) was generated with Benjamini and Hochberg correction. Genes with significant expression change was defined as FC ≥2 and FDR ≤0.05. For sorted healthy donor or RA B cell subsets, STAR 2.5.2a was used to map reads to human genome (HG19). The count data was normalised using rlog implemented in Deseq2. Pooled *t* test was used for group comparison. Significant change was defined as FC >2 and *p-*value <0.05. Pathway analysis was performed using GSEA^72^. Pathway enrichment FDR was generated from *p*-values with Benjamini and Hochberg correction. Other graphing and statistics were performed using R.

### Immuno-histochemical staining of kidney sections

Formalin-fixed paraffin embedded blocks of Kidney needle biopsies form LN patients were purchased from either Conversant Bio or Tissue solutions (*n* = 11, 2 Class II, 3 Class III, 6 class IV). Five-micrometre sections were cut at MedImmune Pathology department and Florescent staining performed on fully automated Ventena autostainer. Slides were loaded onto Ventena, and then went through rehydration and H_2_0_2_ block then antigen retrieval using Cell Conditioning 1. The slides were stained with mouse anti-CD20 clone L26 (Ventena), followed by HRP labelled secondary mouse Omnimap (Ventena) then detected by FITC (Ventena). Slides went through heat denaturation to neutralise any unbound HRP and stripped of the primary Ab using Tris EDTA at 120 degrees for 28 min. The slides then through a 2nd incubation with Rabbit anti-CD11c clone EP134Y (Abcam) followed by HRP labelled Rabbit HQ secondary (Ventena), then detected with Rhodamine (Ventena). Nuclei were stained with DAPI (Molecular probes) and slides mounted using prolong media (Life technologies). Images acquisition using Leica SP5 inverted confocal scope using a 40× oil objective. To quantify infiltration of CD20^+^CD11c^+^ B cells in kidney biopsies, between 1–6 200X section per biopsy was examined. Sections were considered to contain B cell infiltrates if more than 10 DAPI^+^CD20^+^ cells were noted, where the 'B cell-positive' sections contained between 13–118 B cells (mean of 48 B cells per section). The sections that were considered 'B cell-negative', contained between 0 and 8 B cells. These sections were then enumerated for the number of cells positive for DAPI, CD20 and CD11c. If more than one section per biopsy was examined, the mean number of CD11c^+^CD11c^+^ cells/section was used for quantification.

### Statistical analysis

Group comparison was performed using unpaired *t*-test and Welch’s correction was applied as equal variance was not necessarily satisfied (Figs. 3c, 4g, 4h, 8b, 8c, Supplementary Figs. 2b, 3a, 3b, 4d, 10a). Mann–Whitney *U*-test was applied when the values were not normally distributed (Fig. 1b–d, Supplementary Fig. 1b). Correlation was performed using Pearson correlation test (Supplementary Figs. 1c, 4e, 10b). Significance was noted by *p* value: *p < 0.05; **p < 0.01; ***p < 0.001, ****p < 0.0001. The above tests were performed using GraphPad Prism (GraphPad software). Statistical analysis for autoantigen arrays and RNAseq was described above in “Autoantigen arrays” and “RNAseq and data analysis”.

### Study approval

For healthy donors of MedImmune employees, all protocols and informed consent forms were approved by Chesapeake Institutional Review Board (Protocol 2010-001 version 4.0). For lupus and RA donors, the studies were approved by the Institutional Review Board of the National Institute of Arthritis and Musculoskeletal and Skin Diseases (protocol 94-AR-0066, and 00-AR-0222, respectively).

### Data availability

All relevant data are available from the corresponding author on request. The RNAseq data have been deposited in Gene Expression Omnibus (https://www.ncbi.nlm.nih.gov/geo/) under the accession number GSE110999.

## Electronic supplementary material

Supplementary Information
