# Supplementary material for: IL-21 drives expansion and plasma cell differentiation of autoreactive CD11chiT-bet+ B cells in SLE
Source: Nat Commun. 2018 May 1;9:1758. doi: 10.1038/s41467-018-03750-7 (PMC5931508; doi:10.1038/s41467-018-03750-7)
Supplement: Supplementary file 1 — Supplementary Information [file 41467_2018_3750_MOESM1_ESM.pdf]

IL-21 drives expansion and plasma cell differentiation of autoreactive CD11c^{hi}T-bet^{+} B cells in SLE

Wang et al.

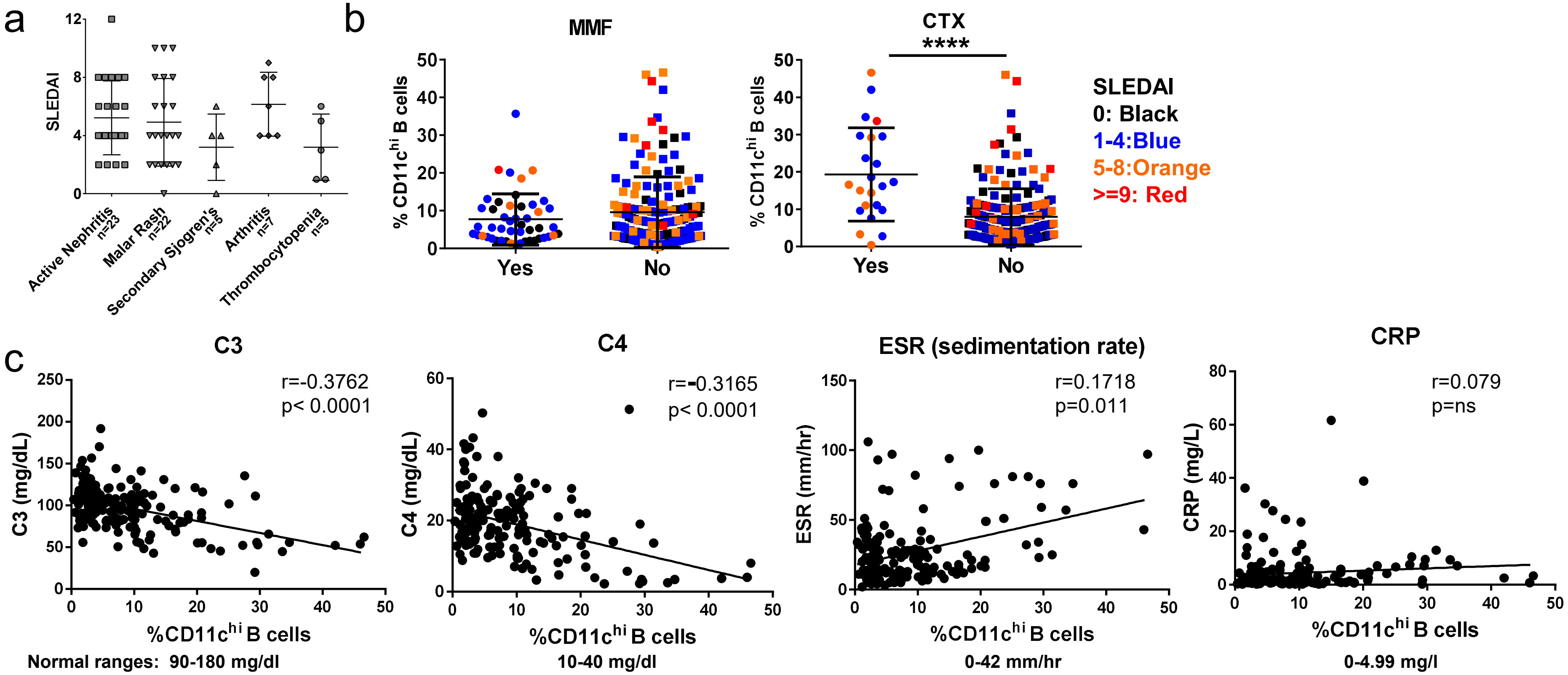

Supplementary Figure 1. CD11c^{hi} B cell correlations to clinical parameters. **(a)** Clinical manifestations in SLE was compared to SLE activity index (SLEDAI). Data shown as mean +/- SEM. **(b)** %CD11c^{hi} B cells (of CD19^{+} cells) was compared to treatment with either Cell Cept (MMF) or cyclophosphamide (CTX), and SLEDAI is indicated. ****p= 0.0001 as determined by Mann-Whitney U-test. Data shown as mean +/- SEM. **(c)** Correlations of %CD11c^{hi} B cells (of CD19^{+} cells) from all SLE donors was compared to disease parameters. (Pearson correlation test).

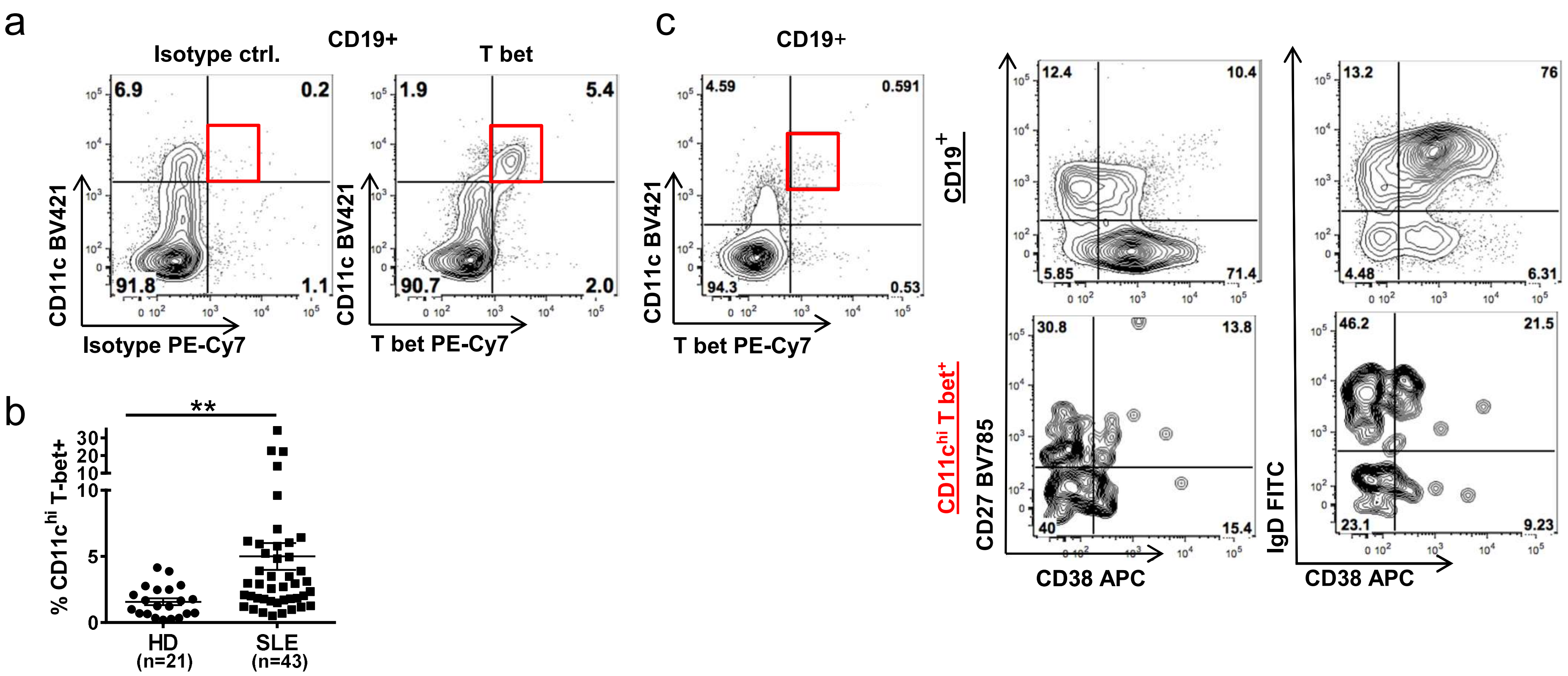

Supplementary Figure 2. CD11c^{hi} T-bet^{+} B cells are significantly expanded in SLE and their phenotype in healthy donors. **(a)** Cell surface phenotype of CD19^{+}CD11c^{hi} T-bet^{+} B cells from SLE donors was compared to isotype-matched (mouse IgG PE-Cy7) negative control. **(b)** Enumeration of %CD11c^{hi} T-bet^{+} (of CD19^{+} cells). Data are represented as mean +/- SEM, unpaired t test with Welch's correction, n= 21 unique healthy donors and 39 unique SLE with 4 repeat SLE donors. **$p<0.01$. **(c)** Cell surface phenotype of CD19^{+}CD11c^{hi} T-bet^{+} B cells was determined from blood of healthy donors. (n=20 unique, with 1 repeat healthy donor(s) examined).

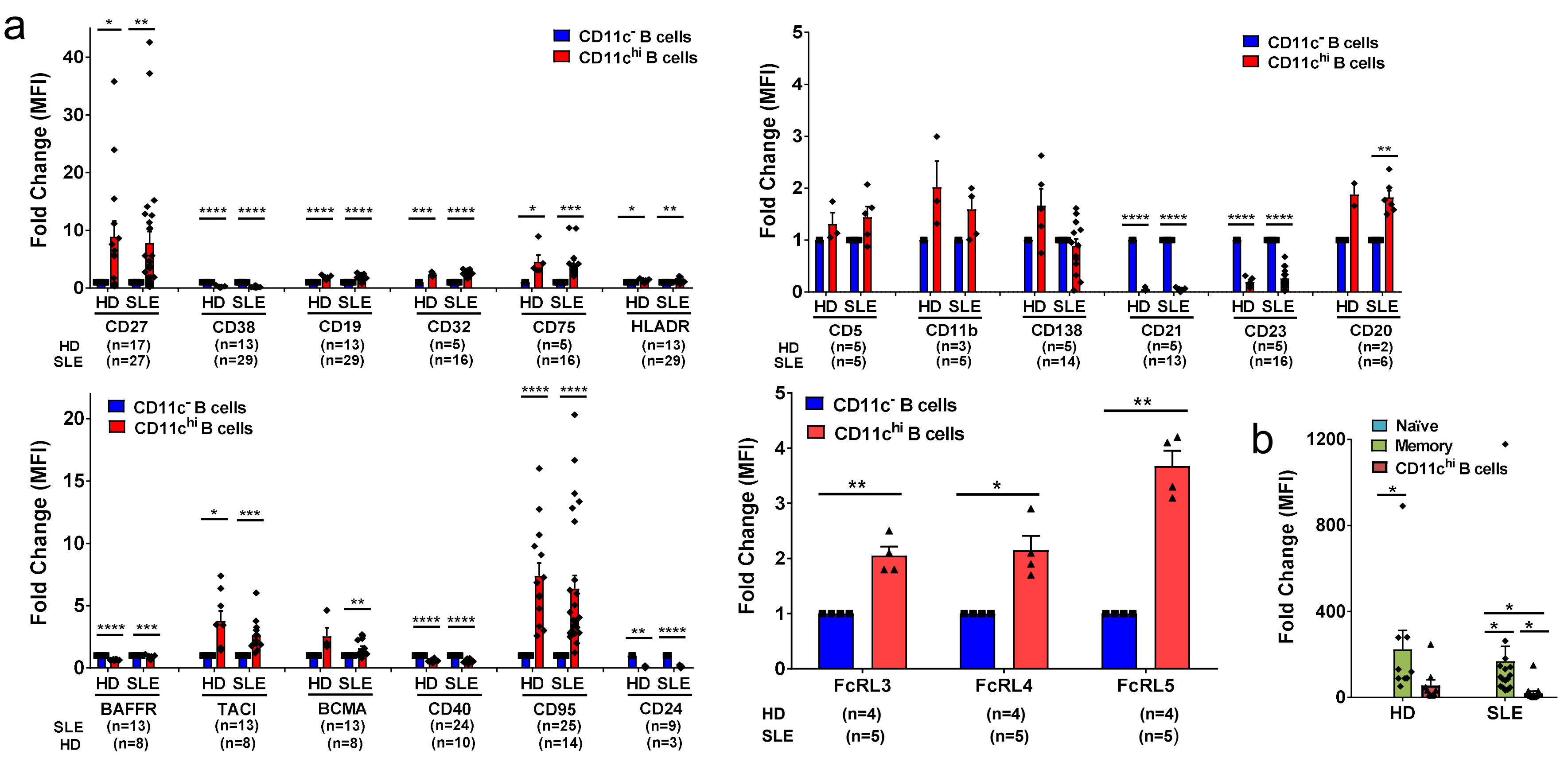

Supplementary Figure 3. Phenotype of CD11c^{hi} B cells from SLE and healthy donors. **(a)** Change in mean fluorescence intensity (MFI) of surface markers expressed by CD19^{+}CD11c^{hi} B cells compared to CD19^{+}CD11c^{-} B cells from healthy donors or SLE donors. **(b)** Change in mean fluorescence intensity (MFI) of CD27 expressed by CD19^{+}CD11c^{hi} B cells and CD19^{+}CD11c^{-}IgD^{-}CD27^{+} memory B cells compared to CD19^{+}CD11c^{-}IgD^{+}CD27^{-} naïve B cells from healthy donors (n=9) or SLE (n=16) donors. Data are represented as mean +/- SEM. Samples with a negative MFI were excluded. *p<0.05, **p<0.01, ***p<0.001, ****p<0.0001 as determined by unpaired T-test with Welch's correction.

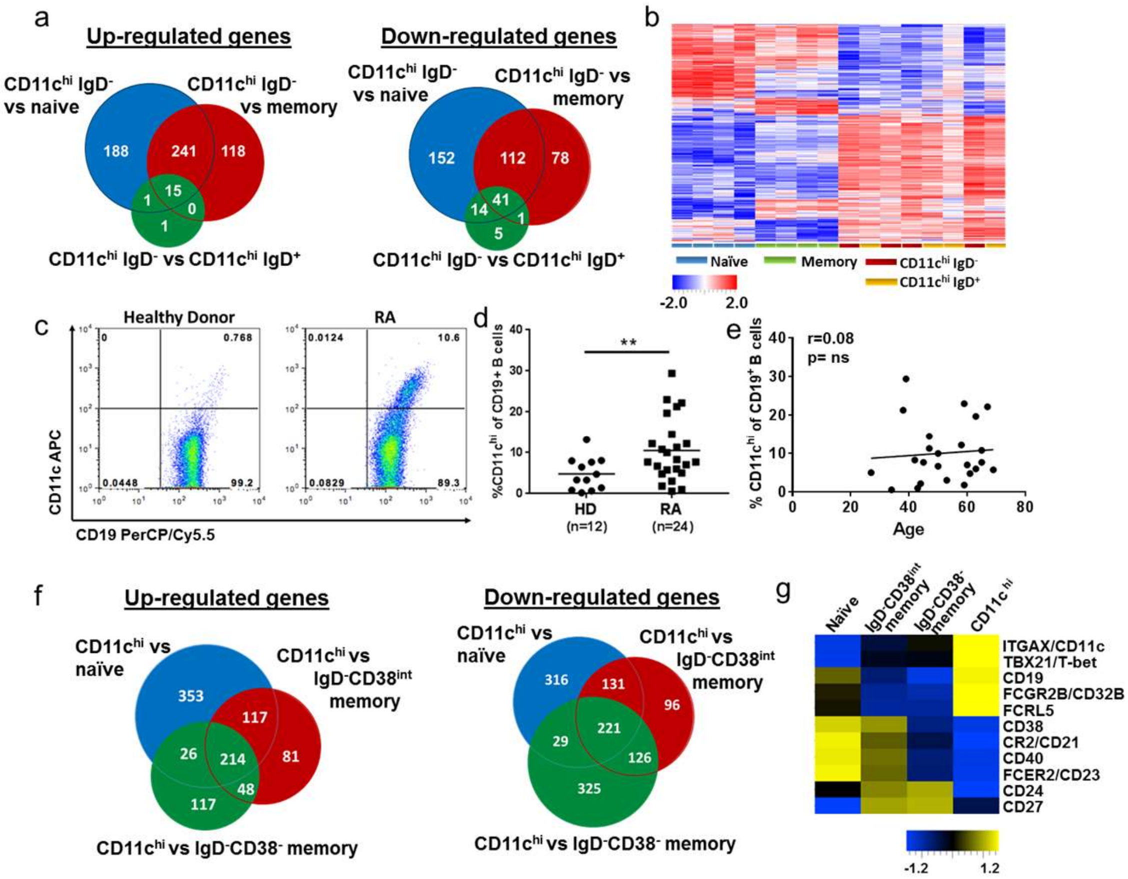

Supplementary Figure 4. Unique transcriptome profile of CD11c^{hi} B cells in SLE and RA. (**a**) SLE: Venn diagram showing overlap of genes with significant change (FC>2, FDR<0.05) in the group comparisons (CD11c^{hi}IgD^{-} vs naïve or CD11c^{hi}IgD^{-} vs memory B cells or CD11c^{hi}IgD^{-} vs CD11c^{hi}IgD^{+} B cells) and passed cutoff of median CPM>50 in at least one of the cell populations. (**b**) SLE: Heatmap showing expression pattern of genes with significant change (FC>2, FDR<0.05) in at least one of the group comparison (CD11c^{hi}IgD^{-} vs naïve or CD11c^{hi}IgD^{-} vs memory B cells or CD11c^{hi}IgD^{-} vs CD11c^{hi}IgD^{+} B cells), and passed cutoff of median counts per million (CPM) >50 in at least one of the cell populations. Log2 transformed CPM values were used for plotting. Genes were clustered using hierarchical clustering. Red indicates higher expression, and blue indicates lower expression. Color bar indicates Z score. (**c**) RA: Representative gating used to define CD11c^{hi} cells (of total CD19^{+} B cells) in a representative healthy (left) or RA (right) donor. (**d**) Quantitation of CD11c^{hi} B cells in healthy donors (n=12) and RA patients (n=24, 20 unique and 4 repeat), as defined in (**c**) unpaired t test with Welch's correction, p=0.006. Data shown as mean +/- SEM(**e**) %CD11c^{hi} B cells in female RA patients (n=23), or male patient (n= 1) was compared to age of individual. r: Pearson correlation coefficient. p= non-significant (ns). (**f**) RA: Venn diagram showing overlap of genes with significant change (FC>2, P value<0.05) in the group comparisons (CD11c^{hi} vs IgD^{-}CD38^{-} memory or CD11c^{hi} vs IgD^{-}CD38^{int} memory B cells or CD11c^{hi} vs IgD^{+}CD38^{int} naïve B cells). (**g**) RA: Heatmap showing expression pattern of representative genes with relevant functions. Average of expression levels for each cell population (n=4 independent samples for each population but naïve B cells where n=3) from RNA sequencing were used for plotting. Yellow indicates higher expression, and blue indicates lower expression. Color bar indicates Z score. **(a, b)** The sorting strategy for the indicated populations are described in Supplementary Fig. 11b for SLE and **(f, g)** Supplementary Fig. 12b for RA

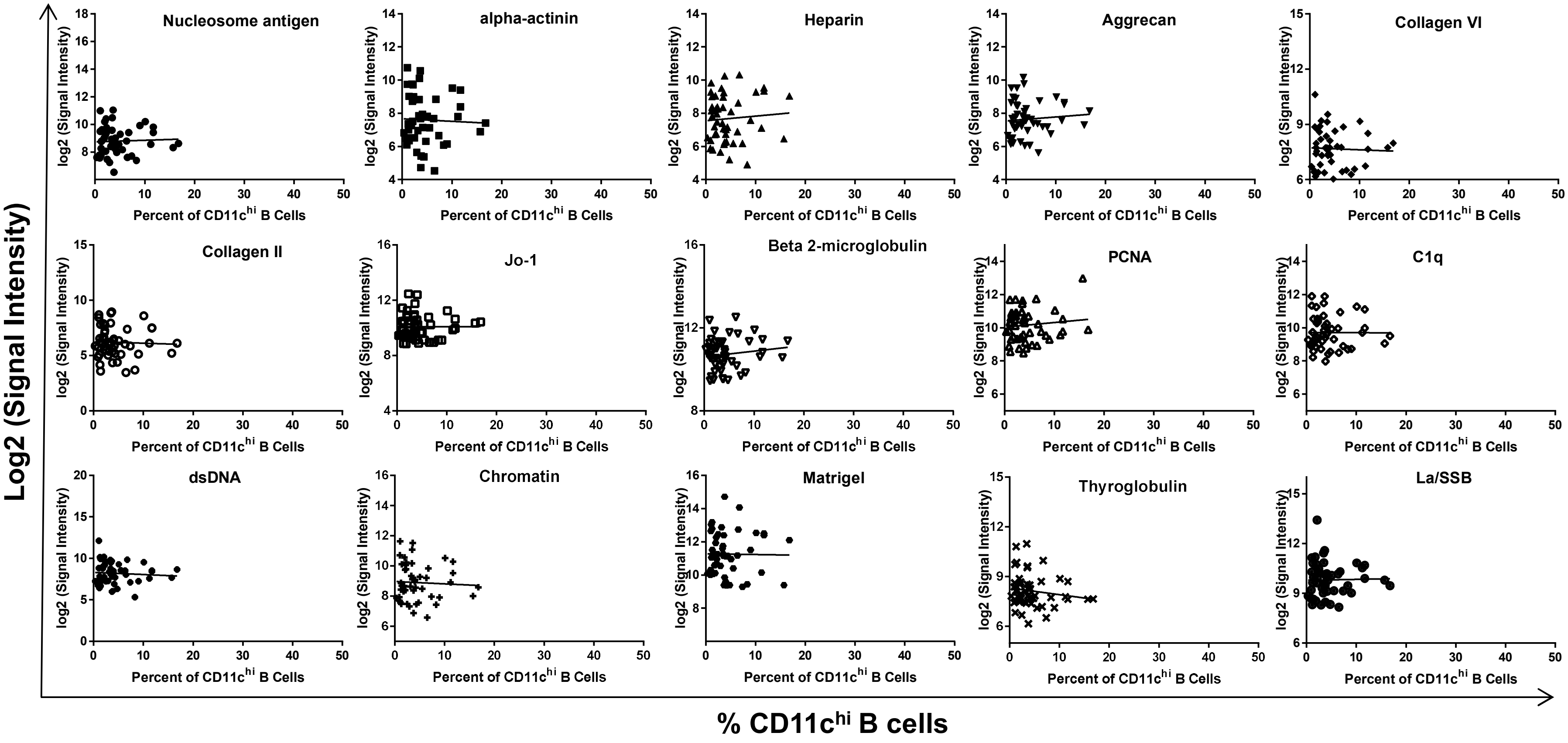

Supplementary Figure 5. Expression of IgG autoantibodies in healthy donors. Scatter plot showing %CD11c^{hi} B cells (of CD19^{+} cells) and log2 (signal intensity) of the serum autoantibodies as in Fig. 6b for healthy donors (n=49 unique donors). None of the 95 autoantibodies tested were found to show significant correlation to %CD11c^{hi} B cells (of CD19^{+} cells) in healthy donors (defined as Pearson correlation test FDR<0.05).

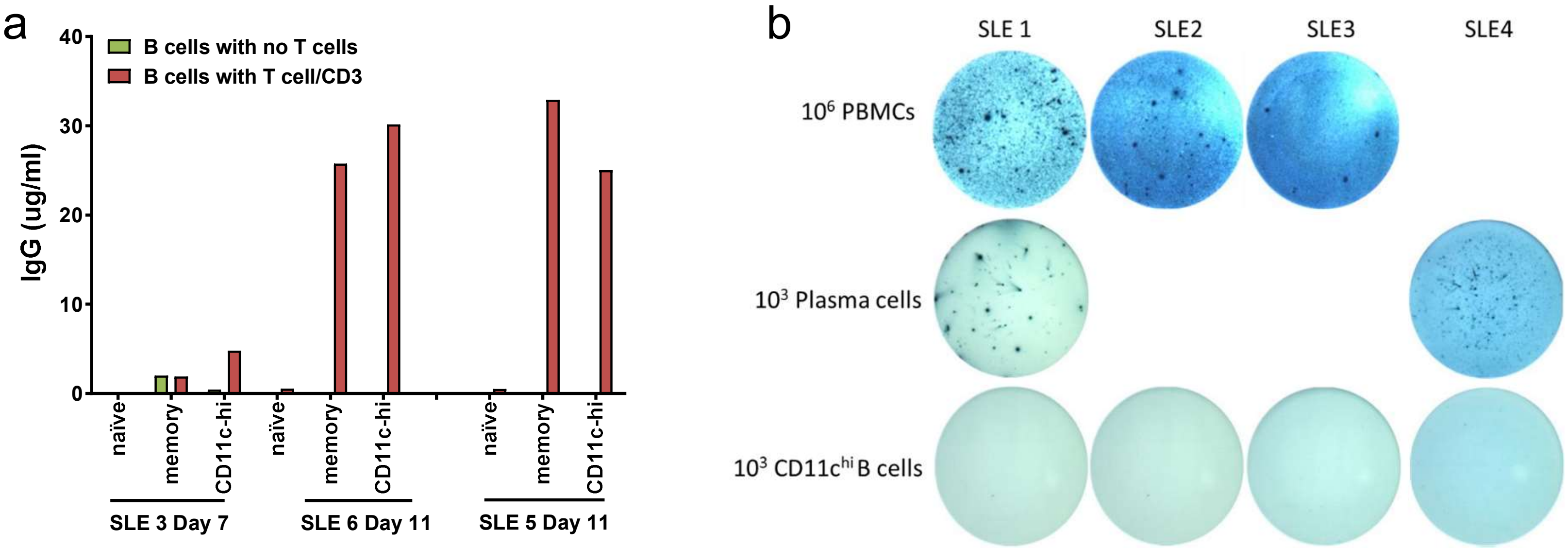

Supplementary Figure 6. CD11c^{hi} B cells do not spontaneously produce IgG. **(a)** Total IgG in the supernatant of CD19^{+} B cell subsets as indicated, cultured in the presence or absence of anti-CD3-activated T cells was determined by ELISA on day noted. **(b)** Total peripheral blood mononuclear cells (PBMC) or sort-purified primary plasma cells (as defined by CD19^{+}CD27^{hi}CD38^{hi}), or CD11c^{hi} B cells (as defined by CD19^{+}CD11c^{hi}) were evaluated for IgG production in a short-term (16 hours) *ex-vivo* ELIspot assay. Each blue dot represents an IgG secreting plasma cell. **(a, b)** The sorting strategy for indicated populations are described in Supplementary Fig. 2a.

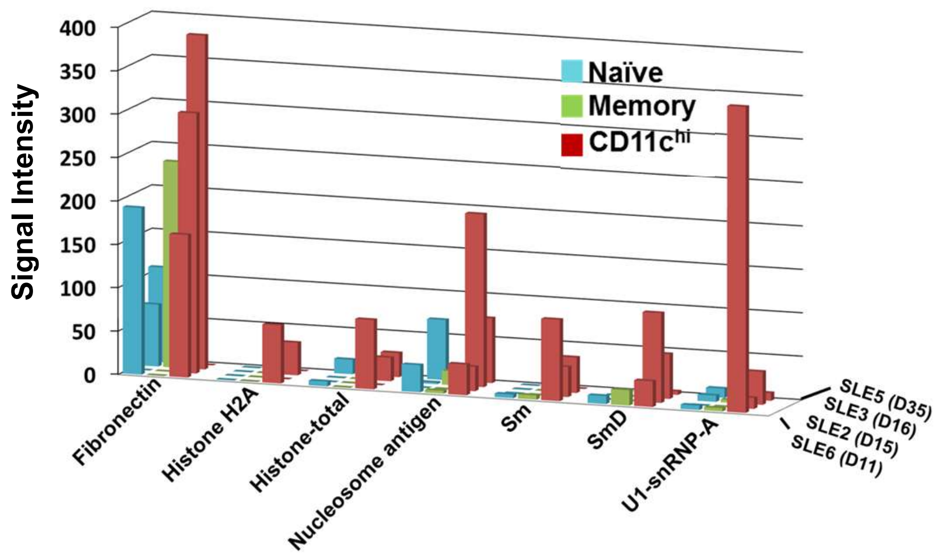

Supplementary Figure 7. CD11c^{hi} B cells produce the majority of autoantibodies. B cells from SLE patients were sorted into CD19^{+}CD11c^{-}CD27^{-} naïve (blue), CD19^{+}CD11c^{-}CD27^{+} memory (green), or CD19^{+}CD11c^{hi} (red) B cells and added to anti-CD3-activated T cells. Autoantibodies in the supernatant were screened for reactivity to 95 self-antigens on culture day indicated. Bar plot shows autoantibody specificities that are significantly increased (FDR≤0.05) in CD11c^{hi} B cells compared to memory B cells as described in Methods. The sorting strategy for the indicated populations are described in Supplementary Fig. 11c.

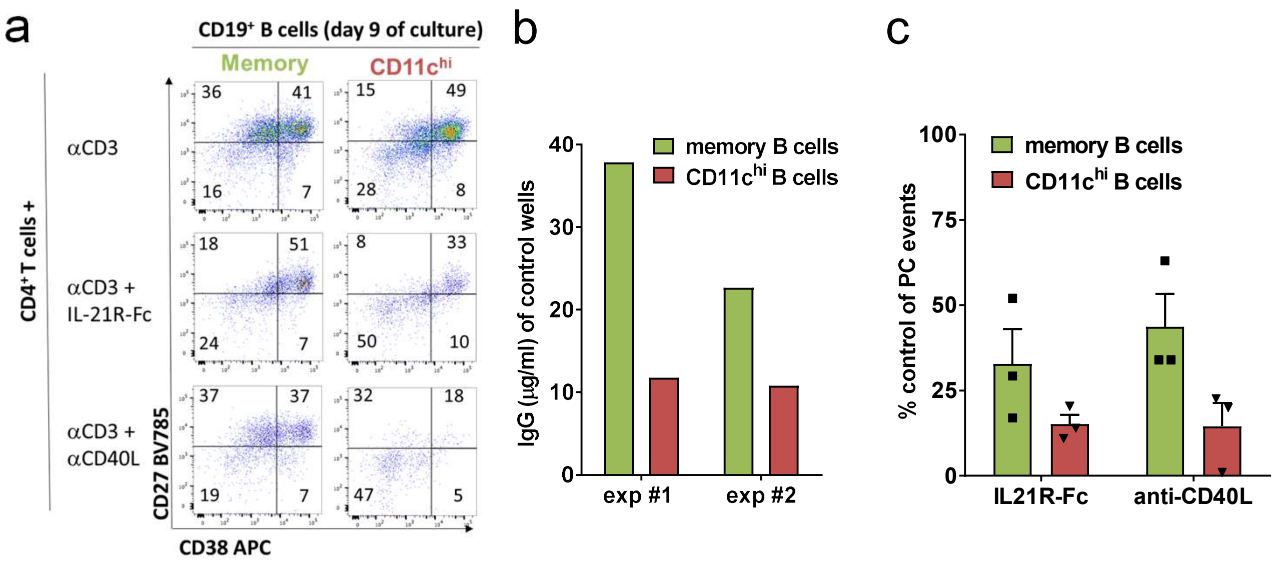

Supplementary Figure 8. CD11c^{hi} B cells from healthy donors also differentiate into plasma cells dependent on IL-21 and CD40L. **(a-d)** B cells from healthy donors were sorted into CD19^{+}CD11c^{-}CD27^{+} memory B cells (green), or CD19^{+}CD11c^{hi} B cells (red) and added to anti-CD3-activated T cells in the presence of either IL-21 or CD40L neutralizing reagents. After 7-9 days of culture, CD19^{+}CD3^{-} B cells were analyzed for CD27^{+}CD38^{hi} plasma cell phenotype, and IgG in the supernatant determined. (**a**) Representative Flow plot of culture conditions shown as indicated. All conditions were collected for the same length of time, thus the number of events displayed is reflective of the relative cell number in the well at time of harvest. (**b**) Quantification of IgG in the supernatant of after 9 days of culture in anti-CD3 control wells without inhibitors added. As the IL-21R-Fc and the anti-CD40L are human IgG1 antibodies, the IgG in these conditions could not be determined. (**c**) The number of plasma cell events collected in anti-CD3 control wells (%100) and compared to the number of events in the IL-21R-Fc or anti-CD40L treated groups. Data shown as mean +/- SEM, n=3 independent experiments. **(a-c)** The sorting strategy for the indicated populations are described in Supplementary Fig. 11c.

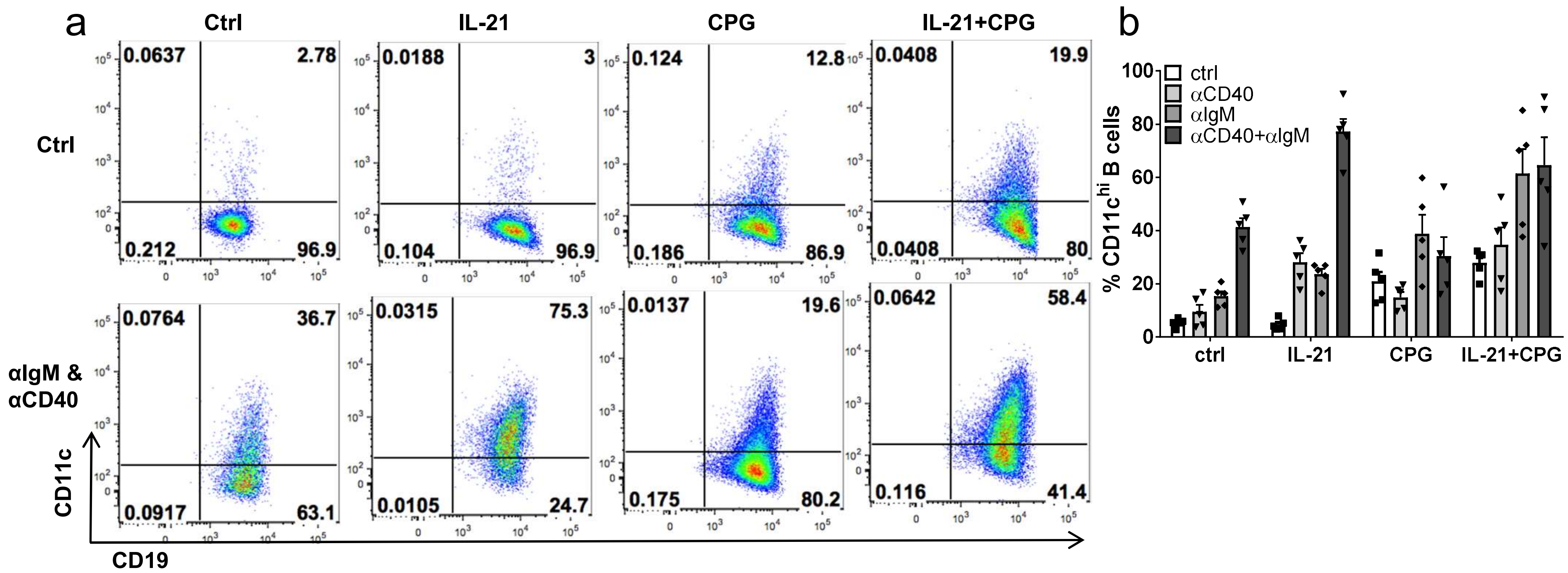

Supplementary Figure 9. IL-21 co-stimulates CD11c expression of naïve B cells. Naïve B cells were isolated from the blood of healthy donors and activated with a combination of stimulators, or control (Ctrl), as indicated. (**a**) Cell surface phenotype of $CD19^+$ B cells was evaluated for CD11c expression after 5 days of culture. Data represents one of 5 independent experiments from 5 unique healthy controls. (**b**) Enumeration (mean +/- SEM) of the frequencies of $CD11c^{hi}$ cells of total $CD19^+$ B cells for each group as indicated from 5 independent experiments.

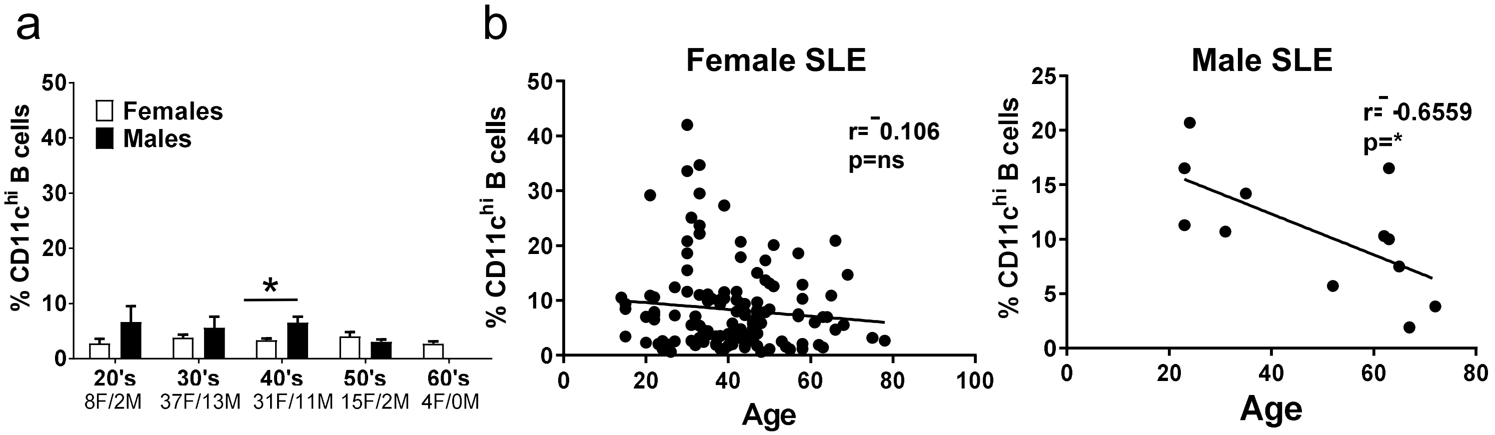

Supplementary Figure 10. Circulating CD11c^{hi} B cells do not increase with age. **(a)** %CD11c^{hi} B cells (of CD19^{+} cells) was determined from blood of healthy donors (number and sex as indicated). Age of healthy donors was provided by decade, (Unpaired t test with Welch's correction, p=0.0173). Data shown as mean +/- SEM. **(b)** %CD11c^{hi} B cells (of CD19^{+} cells) in female SLE patients (n=131), or male patients (n= 12) was compared to age of individual. (Pearson correlation test. r: Pearson correlation coefficient, female SLE: p= non-significant (ns), for male SLE, p=0.021).

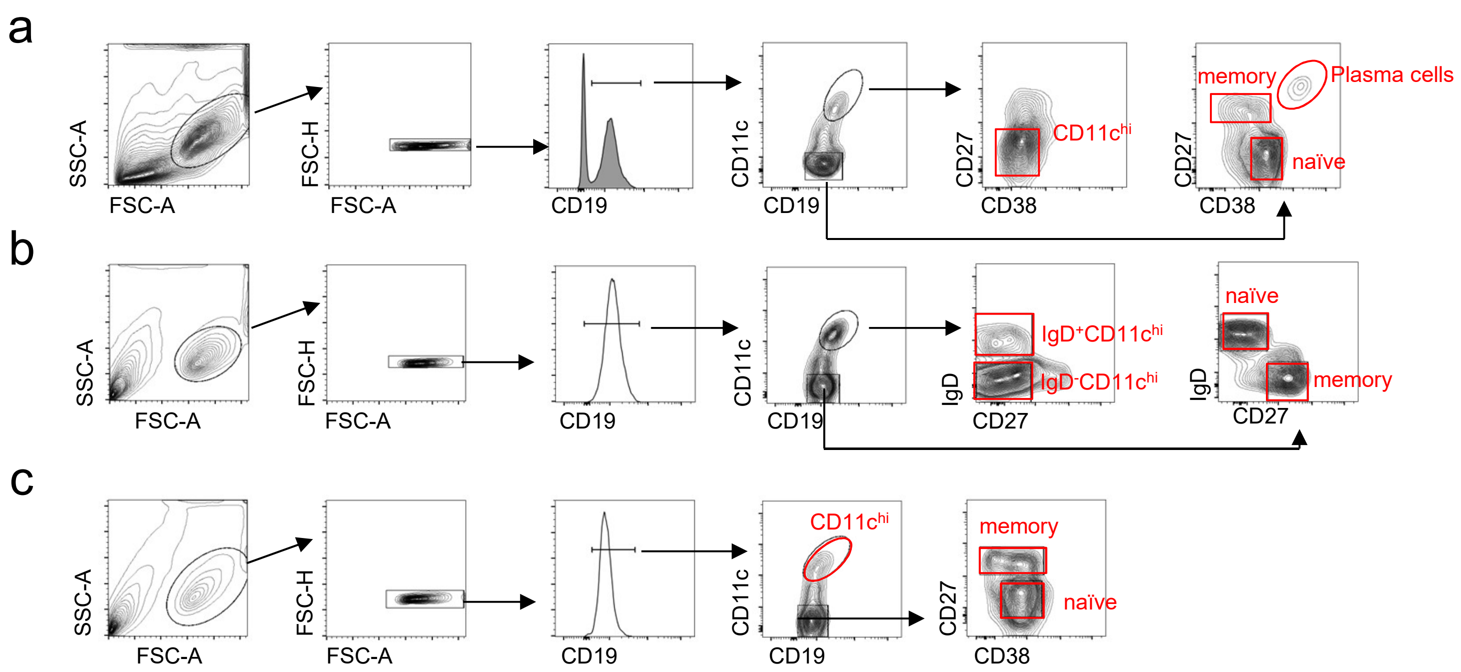

Supplementary Figure 11. Gating strategy used for cell sorting. **(a)** Gating strategy to sort naïve B cells ($CD19^+CD11c^-CD38^+CD27^-$), memory B cells ($CD19^+CD11c^-CD27^+$), plasma cells ($CD19^+CD11c^-CD27^{hi}CD38^{hi}$), or $CD11c^{hi}$ B cells ($CD19^+CD11c^{hi}$ $CD38^-CD27^-$) to determine the telomere length presented in Fig. 4h. **(b)** Gating strategy to sort naïve B cells ($CD19^+CD11c^-IgD^+CD27^-$), memory B cells ($CD19^+CD11c^-IgD^-CD27^+$), IgD^+CD11c^{hi} B cells ($CD19^+CD11c^{hi}IgD^+CD27^-$), or IgD^-CD11c^{hi} B cells ($CD19^+CD11c^{hi}IgD^-CD27^-$) from SLE donors for transcriptome analysis presented on Fig. 5 and Supplementary Fig. 4a, b. **(c)** Gating strategy to sort naïve B cells ($CD19^+CD11c^-CD27\text{-}CD38^+$), memory B cells ($CD19^+CD11c^-CD27^+$), or $CD19^+CD11c^{hi}$ B cells for *in vitro* co-culture experiments presented on Fig. 7 and Supplementary Fig. 7. The same strategy was used to sort naïve B cells on Fig. 8a and $CD11c^{hi}$ B cells and memory B cells on Supplementary Fig. 8a-c.

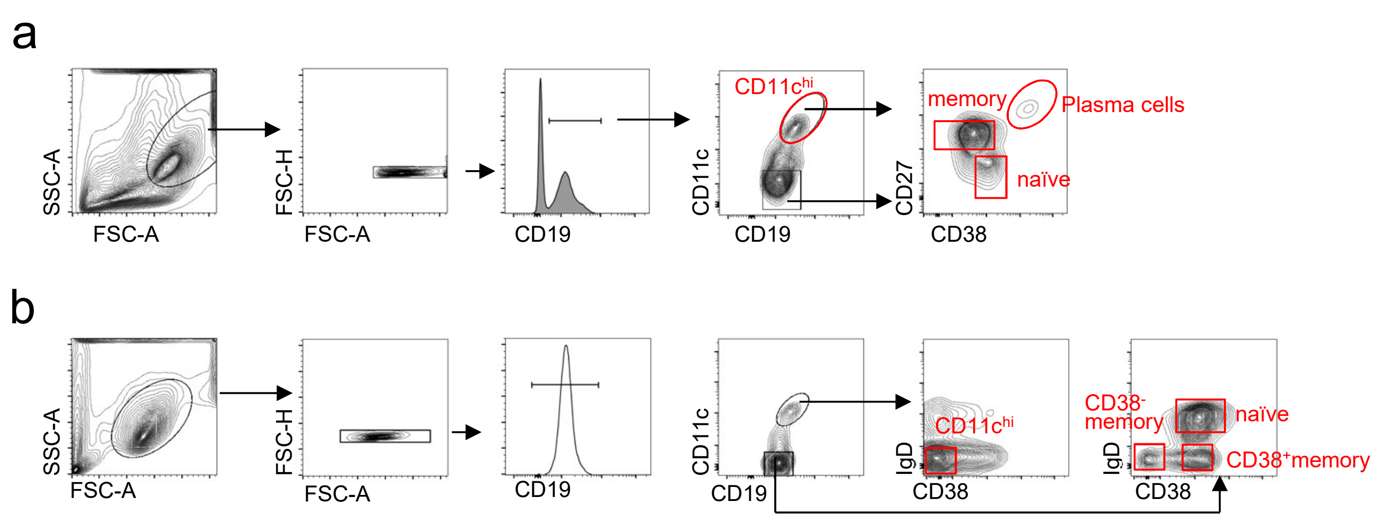

Supplementary Figure 12. Gating strategy used for cell sorting. **(a)** Gating strategy to sort naïve B cells ($CD19^{+}CD11c^{-}CD38^{+}CD27^{-}$), memory B cells ($CD19^{+}CD11c^{-}CD27^{+}$), plasma cells ($CD19^{+}CD11c^{-}CD27^{hi}CD38^{hi}$), or $CD19^{+}CD11c^{hi}$ B cells from SLE donors for ex-vivo ELIspot assay presented in Supplementary Fig. 6a, b. **(b)** Gating strategy to sort naïve B cells ($CD19^{+}CD11c^{-}IgD^{+}CD38^{+}$), $CD38^{+}$ memory B cells ($CD19^{+}CD11c^{-}IgD^{-}CD27^{+}CD38^{+}$), $CD38^{-}$ memory B cells ($CD19^{+}CD11c^{-}IgD^{-}CD27^{+}CD38^{-}$) or $CD11c^{hi}$ B cells ($CD19^{+}CD27^{-}CD38^{-}IgD^{-}CD11c^{hi}$), from healthy donors and RA donors for transcriptome analysis presented in Supplementary Fig. 4f, g.

Supplementary Table 1. The demographics and clinical characteristics of healthy donors and lupus patients examined in this study

	HD[A]		SLE[C]	
	**CD11c^{hi}**	**CD11c^{hi}Tbet^{+}**	**CD11c^{hi}**	**CD11c^{hi}Tbet^{+}**
Total number examined for CD11c phenotype	147	21	221	43
Unique number examined for CD11c phenotype	123	21	112	39
Gender (M/F)	(M=29, F=118)	(M=2, F=19)	(M=12, F=209)	(M=2, F=41)
Age [median (range)]	35 (25 - 65)[B]	45 (35 - 55)[B]	42 (14 - 78)	45 (16 - 78)
SLEDAI [median (range)]	---	---	2 (0 -12)	4 (0 - 10)
C3, mg/dL [median (range)]	---	---	96.1 (20.2 - 191.7)	95.1 (56.6 - 175.1)
C4, mg/dL [median (range)]	---	---	18.5 (2.2 - 51.3)	17.7 (3.9 - 42.6)
ESR mm/hr [median (range)]	---	---	21 (2 - 106)	20 (3 - 76)
CRP mg/L [median (range)]	---	---	1.55 (0.15 - 61.6)	1 (0.2 - 30.3)
Auto-antibodies				
anti-dsDNA (% positive)	---	---	77%	72%
anti-nuclear Abs, ANA (% positive)	---	---	94%	90%
Lupus anti-coagulant, LAC (% positive)	---	---	29%	37%
Extractable nuclear antigens, ENA (% positive)	---	---	85%	84%
Medication				
Glucocoriticoids (number low <10 mg)	---	---	151	31
Glucocoriticoids (number high >15 mg)			35	4
Hydroxychloroquine (number)	---	---	158	31
Azthioprine (number)	---	---	44	12
Cyclophosphamide (number)	---	---	23	2 1
Mycophenolate mofetil (number)	---	---	49	11
Methotrexate (number)	---	---	14	2
Biologics (number, Belimumab/Rituxan)	---	---	4	0

[A]Human whole blood was collected from healthy donors (HD) from the MedImmune Blood Donor program.

[B]Age of the healthy donors was provided by decade.

[C]SLE blood samples were obtained from the National Institute of Arthritis and Musculoskeletal and Skin Diseases/National Institute of Health Clinical parameters for SLE patients were obtained at time of visit by routine laboratory test.

Supplementary Table 2. RA cohort demographics and clinical characteristics

	RA
N examined for CD11c phenotype	24
Unique N examined for CD11c phenotype	20
Gender (M/F)	(M=1, F=23)
Age [mean (range)]	52 (27 - 69)
CRP mg/L [mean (range)]	3.94 (0.02 – 16.8)
anti-CCP (% positive)	66.7%
DMARDs[A,B]	
Methotrexate	87.5%
anti-malarial	70.8%
anti-TNF	58.3%

[A]DMARDs: disease modifying anti-rheumatic drugs
[B]Number of patients on other drugs: abatacept (2); leflunomide (1)

Supplementary Table 3. CD11c^{hi} B cells significantly correlate to a distinct set of IgG autoantibodies

Autoantibody[A]	Specificity	Pearson correlation coefficient	False discovery rate[B]
Nucleosome antigen	Nucleosome antigen	0.433	0
alpha-actinin	Alpha-actinin	0.431	0
Heparin	Heparin	0.425	0
Aggrecan	Aggrecan	0.418	0
Collagen VI	Collagen VI	0.415	0
Collagen II	Collagen II	0.397	0
Jo-1	Histidyl t-RNA synthetase (Jo-1)	0.396	0
Beta 2-microglobulin	Beta-2-Microglobulin (B2M)	0.394	0
PCNA	Proliferating Cell Nuclear Antigen (PCNA)	0.394	0
C1q	C1q	0.393	0
dsDNA	dsDNA	0.392	0
Matrigel	Matrigel	0.389	0
Chromatin	Chromatin	0.388	0
Thyroglobulin	Thyroglobulin (TG)	0.371	0
La/SSB	Lupus La protein (La/SSB)	0.362	0.001
Mitochondrial antigen	Mitochondrial antigen	0.362	0.001
GBM-dissociated	Glomerular Basement Membrane - dissociated (GBM-dissociated)	0.356	0.001
Histone H2A	Histone 2A (H2A)	0.344	0.001
Cytochrome C	Cytochrome C	0.34	0.001
Histone-total	Histone-total	0.337	0.001
LC1	Liver Cytosol Type 1 (LC1)	0.336	0.001
Peroxiredoxin 1	Peroxiredoxin 1	0.334	0.001
ssDNA	ssDNA	0.334	0.001
Elastin	Elastin	0.33	0.001
Collagen I	Collagen I	0.329	0.001
ds RNA	dsRNA	0.329	0.001
Hinstone H4	Histone 4 (H4)	0.307	0.004
Chondroitin Sulfate C	Chondroitin Sulfate C	0.306	0.004
TTG	Tissue Transglutaminase (TTG)	0.305	0.004
Histone H2B	Histone 2B (H2B)	0.303	0.004
PM/Scl-100	Exosome 3'-5' exoribonuclease complex RRP6 (PM/Scl-100)	0.301	0.004
Mi-2	Mi-2	0.295	0.005
LKM1	Cytochrome P450 2D6 (LKM1 hp)	0.294	0.005
Beta 2-glycoprotein I	Beta 2-glycoprotein I	0.283	0.007
CENP-B	Centromere Protein B (CENP-B)	0.282	0.007
Ribo phaspho protein P2	Ribosomal phosphoprotein P2 (RPLP2)	0.268	0.011
Heperan Sulfate	Heperan Sulfate (HS)	0.264	0.012
Fibrinogen IV	Fibrinogen IV	0.261	0.013
Hemocyanin	Hemocyanin	0.261	0.013
Entaktin EDTA	Entaktin EDTA (NID-1)	0.26	0.013
Ribo phaspho protein P1	Ribosomal phosphoprotein P1 (RPLP1)	0.26	0.013
Phophatidylinositol	Phophatidylinositol	0.25	0.018
TPO	Thrombopoietin (TPO)	0.248	0.019
BPI	Bactericidal Permeability Increasing Protein (BPI)	0.241	0.022
Sm	Smith Antigen (Sm)	0.241	0.022
CENP-A	Centromere Protein A (CENP-A)	0.236	0.026
Topoisomerase I	Topoisomerase I	0.23	0.03
Amyloid	Amyloid	0.228	0.031
PL-12	Alanyl-tRNA Synthetase (PL-12)	0.228	0.031
Myosin	Myosin	0.222	0.035
Sm/RNP	Smith Antigen/RNP mixture (Sm/RNP)	0.217	0.04
Histone H1	Histone 1 (H1)	0.216	0.04
Collagen V	Collagen V	0.214	0.042
GP2	Glycoprotein 2 (GP2)	0.21	0.046
U1-snRNP-BB'	U1 Small Nuclear Ribonucleoprotein BB' (U1-snRNP-BB')	0.206	0.049

[A]Of the 95 autoantibodies screened, 55 showed significant correlation with the percentage of CD11c^{hi} B cells.
[B]Significance determined by False discovery rate (FDR) of Pearson correlation test ≤0.05. Table ranked by Pearson correlation coefficient.

Supplementary Table 4. IgG autoantibodies that do not correlate with CD11c^{hi} B cells

Autoantibody[A]	Specificity	Pearson correlation coefficient	False discovery rate[B]
Hinstone H3	Histone 3 (H3)	0.205	0.051
SP100	Speckled 100 KDa Antigen (SP100)	0.203	0.053
Gliadin-IgG	Gliadin-deamidated	0.197	0.062
Fibrinogen S	Fibrinogen S	0.195	0.063
Anti-IgG	Total IgG	0.192	0.066
Decorin-bovine	Decorin-bovine	0.192	0.066
Proteoglycan	Proteoglycan	0.188	0.071
DGPS	1,2-diacyl-sn-glycero3-phospho-L-serine	0.184	0.078
Nucleolin	Nucleolin	0.182	0.079
Ribo phasphoprotein P0	Ribosomal phosphoprotein P0 (RPLP0)	0.182	0.079
Vitronectin	Vitronectin	0.180	0.082
Intrinsic Factor	Intrinsic Factor	0.177	0.087
U1-snRNP-C	U1 Small Nuclear Ribonucleoprotein C (U1-snRNP-C)	0.158	0.132
Heparan HSPG	Heparan Sulfate Proteoglycan (HSPG)	0.154	0.142
M2 Antigen	Mitochondrial 2-oxo acid dehydrogenase (M2)	0.153	0.142
Laminin	Laminin	0.153	0.142
Ro/SSA-52KDa	Lupus Ro Protein 52KDa (Ro/SSA-52KDa)	0.151	0.145
Scl-70	Topoisomerase I 70KDa subunit (Scl-70)	0.151	0.145
MPO	Myeloperoxidase (MPO)	0.144	0.164
SRP54	Signal Recognition Particle 54KDa (SRP54)	0.139	0.179
SmD	Smith Antigen D (SmD)	0.139	0.179
MBP-myelin basic protein	Myelin Basic Protein (MBP)	0.132	0.198
Ro/SSA-60KDa	Lupus Ro Protein 60KDa (Ro/SSA-60KDa)	0.132	0.198
Collagen IV	Collagen IV	0.131	0.202
Vimentin	Vimentin	0.124	0.226
U1-snRNP-A	U1 Small Nuclear Ribonucleoprotein A (U1-snRNP-A)	0.121	0.234
Nup62	Nucleoporin 62kDa (Nup62)	0.118	0.247
Collagen III	Collagen III	0.117	0.249
KU -P70/P80	ATP-dependent DNA helicase II 70/80 kDa subunit (KU -P70/P80)	0.114	0.261
PM/Scl-75	Exosome complex component RRP45 (PM/Scl-75)	0.108	0.282
Fibronectin	Fibronectin	0.103	0.303
PL-7	Threonyl-tRNA Synthetase (PL-7)	0.095	0.342
PR3	Proteinase 3 (PR3)	0.084	0.404
Prothrombin protien	Prothrombin protien	0.078	0.440
Glycated Albumin-human	Glycated Albumin-human	0.074	0.454
U1-snRNP-68	U1 Small Nuclear Ribonucleoprotein 68KDa (U1-snRNP-68)	0.060	0.550
Sphingomyelin	Sphingomyelin	0.058	0.557
gP210	Glycoprotein 210 (gP210)	0.033	0.728
Cardolipin	Cardiolipin	-0.046	0.636
MAG	Myelin-associated Glycoprotein-FC (MAG)	-0.195	0.063

[A]Of the 95 autoantibodies screened, 40 do not show significant correlation with the percentage of CD11c^{hi} B cells.
[B]Significance determined by False discovery rate (FDR) of Pearson correlation test ≤0.05. Table ranked by Pearson correlation coefficient.

Supplementary Table 5. Cell counts for Supplementary Figure 8

	$CD27^+CD38^{hi}$ plasma cell (#)							
	Day 0		Day 7-9 of co-culture with CD4+ T cells					
			αCD3		αCD3+IL21R Fc		αCD3+αCD40L	
Sample	memory*	$CD11c^{hi}$*	memory¥	$CD11c^{hi}$¥	memory¥	$CD11c^{hi}$¥	memory¥	$CD11c^{hi}$¥
HD 1	0	0	8821	2381	2585	485	5559	538
HD 2	0	0	24944	11459	4156	1226	8568	2346
HD 3	0	0	6960	9331	3613	1300	2360	108

*Number (#) of $CD27^+CD38^{hi}$ plasma cells present in total cultured B cells (sorted memory or $CD11c^{hi}$ B cells from healthy donors) at Day 0.

¥Number (#) of $CD27^+CD38^{hi}$ plasma cells present in memory or $CD11c^{hi}$ B cells from healthy donors after 7-9 days co-culture with anti-CD3-activated T cells in the presence of either IL-21 or CD40L neutralizing reagents was determined by flow cytometry.

Supplementary Table 6. Cell counts for Figure 8

	$CD19^{+}CD11c^{hi}$					
	**Day 0**		**Day 5 of culture**			
Sample	(%)*	(#)¥	ctrl.(#)¶	IL21 (#)¶	αIgM/CD40 (#)¶	IL21/αIgM/CD40 (#)¶
SLE 1	0	0	31	57	614	2067
SLE 2	0	0	53	N/A	323	1911
SLE 3	0	0	139	369	5387	14063
SLE 4	0.2	200	379	401	3193	12424
SLE 5	0.2	200	453	557	1646	10531
SLE 6	0	0	146	156	1167	4437

*$CD19^{+}CD11c^{-}CD27^{-}$ naïve B cells were sorted from SLE donors and post sort purity ($\%CD11c^{hi}$ cells) was determined by flow cytometry.

¥Number (#) of $CD11c^{hi}$ B cells present in 100,000 total cultured B cells/well at Day 0 was determined.

¶ Number (#) of $CD19^{+}CD11c^{hi}$ B cells was determined per well after Day 5 of culture by flow cytometry.

Supplementary Table 7. Antibodies used in this study for Flow cytometry

Antigen	Clone	Fluorochrome	µl/1x10⁶ cells	Company
CD5	UCHT2	AF700	5	BD Biosciences
CD11b	ICRF44	BUV395	5	BD Biosciences
CD11c	B-ly6	BV421	5	BD Biosciences
CD11c	B-ly6	PE	5	BD Biosciences
CD19	HIB19	PercPCy5.5	10	Biolegend
CD20	2H7	AF700	5	Biolegend
CD21	Bu32	APC	5	Biolegend
CD23	EBVCS-5	FITC	5	Biolegend
CD24	ML5	PE-Cy7	1	Biolegend
CD27	O323	BV785	10	Biolegend
CD32	AT10	PE	2	Invitrogen
CD38	HB7	APC	1	BD Biosciences
CD38	HB7	BUV395	10	BD Biosciences
CD40	5C3	BUV395	10	BD Biosciences
CD45	HI30	V500	4	BD Biosciences
CD75	LN1	FITC	20	BD Biosciences
CD95	DX2	PE	4	Biolegend
CD138	MI15	PE-Cy7	5	Biolegend
CD307c/FcRL3	H5	BB515	5	BD Biosciences
CD307d/FcRL4	413D12	PE-Cy7	5	Biolegend
CD307e/FcRL5	509f6	APC	5	Biolegend
BAFFR	11C1	PE-Cy7	5	BD Biosciences
BCMA	19F2	PE	5	BD Biosciences
CXCR3	1C6/CXCR3	PE	6	BD Biosciences
HLADR	L243	AF700	4	Biolegend
IgA	IS11-8E10	APC	10	Miltenyi Biotec
IgD	1A6-2	FITC	5	BD Biosciences
IgG	G18-145	PE-Cy7	20	BD Biosciences
IgM	MHM-88	PE	2	Biolegend
IL-21R	2G1-K12	PE	10	Biolegend
TACI	1A1	APC	5	BD Biosciences
T-bet	4B10	PE-Cy7	0.5	eBioscience
Mouse IgG1k Isotype Control	P3.6.2.8.1	PE-Cy7	2	eBioscience

Supplementary note 1. SLE data set

SLE Category	Symbol	Median counts per million (CPM)			CD11c^{hi} (IgD$^-$) vs Naïve			CD11c^{hi} (IgD$^-$) vs Memory		
		Naïve	Memory	CD11c^{hi}(IgD$^-$)	FDR	Fold Change	Significance	FDR	Fold Change	Significance
CD11c^{hi} Phenotype	CD19	542.974	398.089	1137.969	0.000	2.164	TRUE	0.000	2.895	TRUE
	MS4A1/CD20	3914.844	3043.445	5066.821	0.002	1.544	FALSE	0.000	1.799	FALSE
	ITGAX/CD11c	1.288	47.593	1548.528	0.000	1029.986	TRUE	0.000	38.404	TRUE
	TBX21/T-bet	3.319	20.323	109.554	0.000	31.205	TRUE	0.000	6.245	TRUE
	FCRL5	168.246	95.020	1678.000	0.000	10.357	TRUE	0.000	16.276	TRUE
	FCGR2B/ CD32B	74.049	62.179	145.692	0.000	2.034	TRUE	0.000	2.439	TRUE
	CD27	0.835	34.937	2.136	0.000	2.963	TRUE	0.000	0.077	TRUE
	CR2/CD21	142.638	58.316	2.949	0.000	0.016	TRUE	0.000	0.036	TRUE
	FCER2/CD23	434.780	78.529	14.029	0.000	0.024	TRUE	0.000	0.149	TRUE
	CD24	105.433	139.489	8.303	0.000	0.069	TRUE	0.000	0.041	TRUE
	CD38	16.051	6.075	0.929	0.000	0.072	TRUE	0.000	0.228	TRUE
	CD40	218.988	152.946	57.755	0.000	0.282	TRUE	0.000	0.378	TRUE
	FAS/CD95	9.727	35.553	55.314	0.000	6.265	TRUE	0.026	1.917	FALSE
Activation	CD44	313.451	353.130	118.472	0.000	0.410	TRUE	0.000	0.356	TRUE
	CD70	0.685	21.518	2.139	0.000	4.274	TRUE	0.000	0.123	TRUE
	CD72	195.654	36.879	372.292	0.000	1.725	FALSE	0.000	8.563	TRUE
	CD80	2.300	23.441	27.349	0.000	10.078	TRUE	0.915	1.134	FALSE
	CD86	19.502	50.990	146.434	0.000	8.017	TRUE	0.000	3.491	TRUE
	TLR4	3.147	5.703	0.431	0.000	0.180	TRUE	0.000	0.083	TRUE
	TLR8	0.392	0.271	2.123	0.000	5.909	TRUE	0.000	6.774	TRUE
	TLR9	5.004	11.185	21.495	0.000	4.478	TRUE	0.000	1.838	FALSE
	TLR7	66.943	48.374	65.071	0.882	1.062	FALSE	0.000	1.674	FALSE
BCR signaling	SYK	484.494	456.145	1117.112	0.000	2.420	TRUE	0.000	2.528	TRUE
	CARD6	3.940	1.434	7.407	0.000	2.000	TRUE	0.000	5.890	TRUE
	DAPP1	137.024	109.404	452.972	0.000	3.657	TRUE	0.000	4.444	TRUE
	CD79B	972.932	467.585	294.321	0.000	0.306	TRUE	0.000	0.626	FALSE
Co inhibition	SIGLEC6	1.956	20.088	143.329	0.000	79.126	TRUE	0.000	7.495	TRUE
	CTLA4	0.000	0.011	2.087	0.000	79.130	TRUE	0.000	34.444	TRUE
	CD274/PD1	0.884	3.844	11.821	0.000	14.188	TRUE	0.000	3.260	TRUE
	PDCD1/PDL1	0.068	0.728	14.757	0.000	148.860	TRUE	0.000	19.576	TRUE
	LILRB2/CD85a	2.442	3.407	140.336	0.000	81.247	TRUE	0.000	58.169	TRUE
Cytokines/ Receptors	IL6	31.108	24.084	0.950	0.000	0.026	TRUE	0.000	0.037	TRUE
	IL10	0.199	1.145	8.415	0.000	36.498	TRUE	0.000	6.076	TRUE
	LTA	8.047	10.561	3.593	0.000	0.446	TRUE	0.000	0.351	TRUE
	LTB	824.206	924.084	164.586	0.000	0.162	TRUE	0.000	0.133	TRUE
	IL21R	35.585	0.495	34.530	1.000	0.978	FALSE	0.000	71.054	TRUE
	IL4R	1775.318	86.534	78.452	0.000	0.042	TRUE	0.464	0.792	FALSE
	IL6R	0.481	21.235	4.229	0.000	8.255	TRUE	0.000	0.192	TRUE
	IL2RA	4.104	32.801	0.512	0.000	0.123	TRUE	0.000	0.014	TRUE
	IL2RB	0.034	1.299	61.274	0.000	1349.605	TRUE	0.000	24.900	TRUE
	IL13RA1	104.717	62.981	9.268	0.000	0.084	TRUE	0.000	0.163	TRUE
	TNFRSF1B/ TNFR2	75.373	116.286	1053.663	0.000	14.713	TRUE	0.000	9.638	TRUE
	TNFRSF13B/ TACI	12.955	129.974	97.506	0.000	8.681	TRUE	0.379	0.806	FALSE
	TNFSF14/ LIGHT	0.025	0.093	0.383	0.000	11.249	TRUE	0.016	3.554	TRUE
	TNFRSF19/ TROY	2.530	0.299	9.426	0.000	5.624	TRUE	0.000	29.740	TRUE
Differentiation	XBP1	47.184	43.954	67.951	0.034	1.430	FALSE	0.178	1.301	FALSE
	AICDA/AID	0.051	0.242	7.647	0.000	70.522	TRUE	0.000	21.775	TRUE
	BCL6	126.258	14.499	153.633	0.322	1.202	FALSE	0.000	11.476	TRUE
	PRDM1/BLIMP1	7.353	16.523	35.333	0.000	4.685	TRUE	0.330	1.465	FALSE
PC associated	BMP6	0.176	0.318	1.040	0.003	6.211	TRUE	0.241	2.447	FALSE
	EMP3	177.188	273.079	775.089	0.000	4.310	TRUE	0.000	3.045	TRUE
	S100A4	22.590	114.125	80.174	0.000	3.602	TRUE	0.205	0.722	FALSE
Fc Receptors	FCRL1	1208.391	462.150	293.786	0.000	0.257	TRUE	0.002	0.688	FALSE
	FCRL2	325.578	270.203	620.280	0.000	1.894	FALSE	0.000	2.229	TRUE
	FCRL3	439.840	171.879	1098.890	0.000	2.948	TRUE	0.000	6.790	TRUE
	FCRL4	0.119	0.366	12.098	0.000	67.306	TRUE	0.000	31.764	TRUE
	FCGR2A/ CD32A	1.630	10.276	28.441	0.000	16.881	TRUE	0.000	2.797	TRUE

	FCGR2C	10.011	41.126	122.282	0.000	14.187	TRUE	0.000	3.067	TRUE
	FCGR3A/ CD16A	0.019	0.023	1.535	0.000	202.201	TRUE	0.000	162.268	TRUE
	FCGR3B/ CD16B	0.000	0.000	0.222	0.000	26.260	TRUE	0.000	16.342	TRUE
**Migration**	ITGB2/CD18 (LFA1/Mac1 subunit	276.930	188.359	1111.259	0.000	4.108	TRUE	0.000	6.685	TRUE
	ITGB7	121.479	346.724	743.718	0.000	5.682	TRUE	0.000	2.125	TRUE
	ITGAM/CD11b	22.613	193.215	75.178	0.000	3.933	TRUE	0.000	0.415	TRUE
	ITGAD	0.000	0.011	5.755	0.000	2104.912	TRUE	0.000	464.246	TRUE
	CCR7	346.818	145.789	11.109	0.000	0.043	TRUE	0.000	0.097	TRUE
	CXCR4	5566.364	1680.156	631.919	0.000	0.113	TRUE	0.000	0.314	TRUE
	CXCR5	375.230	264.725	31.629	0.000	0.064	TRUE	0.000	0.083	TRUE
	CXCR3	1.288	34.433	10.500	0.000	9.819	TRUE	0.000	0.354	TRUE
	CX3CR1	0.038	0.011	0.141	0.000	10.692	TRUE	0.000	11.526	TRUE
	CCR9	1.604	2.478	3.304	0.230	1.694	FALSE	1.000	1.026	FALSE
	CCR10	1.012	2.318	1.521	0.478	1.385	FALSE	0.095	0.533	FALSE
	CCR6	61.306	27.061	38.549	0.000	0.645	FALSE	0.013	1.312	FALSE
	CCR2	0.356	3.291	0.296	0.405	0.613	FALSE	0.000	0.070	TRUE
	CCR5	0.023	0.680	0.504	0.071	4.277	FALSE	0.549	0.525	FALSE
	CCR1	0.025	1.809	0.105	0.504	2.171	FALSE	0.036	0.202	TRUE
**Transcription factors/regulators**	AIRE	0.763	0.316	23.304	0.000	29.757	TRUE	0.000	45.156	TRUE
	BCL11B	0.000	0.039	28.760	0.000	9569.382	TRUE	0.000	629.468	TRUE
	HOXB7	0.126	1.287	3.934	0.000	30.310	TRUE	0.000	3.251	TRUE
	SOX4	23.010	7.365	37.966	0.001	2.347	TRUE	0.000	6.784	TRUE
	SOX5	2.877	3.574	64.755	0.000	25.197	TRUE	0.000	18.667	TRUE
	FOXP4	86.376	53.499	298.589	0.000	3.299	TRUE	0.000	5.336	TRUE
	FOXF1	0.000	0.000	2.032	0.000	50.623	TRUE	0.000	581.734	TRUE
	POU3F3	0.000	0.000	1.417	0.000	560.831	TRUE	0.000	92.489	TRUE
	PBX4	0.217	3.968	8.515	0.000	36.017	TRUE	0.002	1.919	FALSE
	ADARB1	176.458	40.855	239.221	0.061	1.395	FALSE	0.000	6.599	TRUE
	TFEC	5.295	155.421	270.584	0.000	42.425	TRUE	0.333	1.550	FALSE
	TOX	0.404	20.616	126.692	0.000	248.286	TRUE	0.000	6.348	TRUE
**Signaling**	STAT2	281.970	211.532	134.823	0.000	0.436	TRUE	0.000	0.575	FALSE
	SOCS2	0.104	1.769	0.551	0.000	7.343	TRUE	0.302	0.595	FALSE
	SOCS5	46.800	31.342	21.398	0.000	0.460	TRUE	0.001	0.702	FALSE
	LCK	18.168	11.584	0.314	0.000	0.017	TRUE	0.000	0.022	TRUE
	LAG3	0.011	0.096	0.464	0.000	76.440	TRUE	0.000	9.053	TRUE
	ZAP70	14.451	5.006	42.800	0.000	3.147	TRUE	0.000	8.950	TRUE
	PTPRS	3.246	2.188	149.152	0.000	52.439	TRUE	0.000	48.301	TRUE
	DUSP4/MPK2	0.720	5.436	93.122	0.000	83.489	TRUE	0.000	16.051	TRUE
	DUSP5	247.701	66.604	342.013	0.003	1.871	FALSE	0.000	5.910	TRUE
	FGR	207.427	59.386	1382.569	0.000	8.619	TRUE	0.000	24.198	TRUE
	PRKCH	1.781	1.393	41.011	0.000	22.817	TRUE	0.000	26.241	TRUE
	DLL1	8.059	5.318	28.144	0.000	3.059	TRUE	0.000	4.995	TRUE
	SLAMF7	1.664	16.670	105.942	0.000	65.957	TRUE	0.000	6.461	TRUE
	TCL1A	775.602	5.143	1.925	0.000	0.003	TRUE	0.207	0.622	FALSE
**Other**	TOX2	0.176	1.387	55.717	0.000	377.252	TRUE	0.000	36.907	TRUE
	CD200	64.192	3.335	5.824	0.000	0.094	TRUE	0.011	2.161	TRUE
	S1PR5	0.000	0.000	6.041	0.000	2137.841	TRUE	0.000	472.774	TRUE
	GAS7	14.631	4.397	265.129	0.000	20.996	TRUE	0.000	61.533	TRUE
	ADAM15	19.902	16.911	150.868	0.000	7.132	TRUE	0.000	8.105	TRUE
	SRGN	27.187	36.566	233.420	0.000	10.697	TRUE	0.000	7.626	TRUE
	PRF1	0.000	0.346	1.323	0.000	501.892	TRUE	0.000	4.475	TRUE
	MMP11	13.523	8.794	0.744	0.000	0.062	TRUE	0.000	0.097	TRUE
	MMP17	107.016	1.164	6.464	0.000	0.105	TRUE	0.000	8.156	TRUE
	MS4A7	23.332	12.994	99.093	0.000	4.264	TRUE	0.000	7.821	TRUE
	NKG7	0.173	0.473	61.845	0.000	263.725	TRUE	0.000	126.532	TRUE
	CD5	7.183	1.080	1.524	0.001	0.304	TRUE	0.501	1.446	FALSE

Genes with significant change are indicated (FC>2, FDR<0.05)

Naïve B cells: CD19$^+$ CD11c$^-$ IgD+ CD27$^-$

Memory B cells: D19$^+$ CD11c$^-$ IgD$^-$ CD27$^+$

CD11c^{hi} B cells: CD19$^+$ CD11c$^+$

Supplementary note 2. RA data set

RA		Average expression				CD11c^{hi} vs Naïve			CD11^{hi} vs IgD-CD38- memory			CD11chi vs IgD^{-}CD38^{int} memory		
Category	Symbol	IgD^{+} CD38^{int} naïve	IgD^{-} CD38^{-} memory	IgD^{-} CD38^{int} memory	IgD- CD38- CD11c^{hi}	P value	Fold Change	Significance	P value	Fold Change	Significance	P value	Fold Change	Significance
CD11c^{hi} Phenotype	CD19	8.657	7.515	7.936	9.189	0.391	1.446	FALSE	0.011	3.191	TRUE	0.050	2.382	TRUE
	ITGAX/CD11c	2.619	5.798	4.777	9.210	0.000	96.441	TRUE	0.000	10.647	TRUE	0.000	21.597	TRUE
	TBX21/T-bet	1.790	3.724	3.631	6.410	0.000	24.585	TRUE	0.000	6.438	TRUE	0.000	6.862	TRUE
	FCRL5	9.117	7.893	7.869	10.997	0.008	3.682	TRUE	0.000	8.601	TRUE	0.000	8.746	TRUE
	FCGR2B/CD32B	7.269	6.581	6.667	8.166	0.143	1.862	FALSE	0.013	3.001	TRUE	0.018	2.826	TRUE
	CD27	2.160	4.295	4.238	3.072	0.047	1.882	FALSE	0.010	-2.333	TRUE	0.013	-2.243	TRUE
	CR2/CD21	7.716	5.888	6.863	4.829	0.002	-7.394	TRUE	0.227	-2.083	FALSE	0.025	-4.093	TRUE
	FCER2/CD23	8.596	5.262	7.258	4.125	0.000	-22.178	TRUE	0.066	-2.200	FALSE	0.000	-8.775	TRUE
	CD24	7.224	8.414	8.143	5.209	0.001	-4.039	TRUE	0.000	-9.217	TRUE	0.000	-7.638	TRUE
	CD38	5.373	2.613	4.847	1.743	0.000	-12.384	TRUE	0.046	-1.828	FALSE	0.000	-8.596	TRUE
	CD40	8.879	7.881	8.535	7.553	0.031	-2.507	TRUE	0.579	-1.255	FALSE	0.103	-1.975	FALSE
Activation	CD72	8.066	5.265	6.224	8.346	0.666	1.214	FALSE	0.000	8.460	TRUE	0.003	4.354	TRUE
	TLR9	4.705	5.452	5.356	6.362	0.002	3.153	TRUE	0.071	1.879	FALSE	0.047	2.009	TRUE
	CD70	1.672	3.662	3.351	2.140	0.330	1.384	FALSE	0.003	-2.871	TRUE	0.016	-2.315	TRUE
Co inhibition	LILRB2/CD85a	2.987	3.247	3.819	6.730	0.000	13.389	TRUE	0.000	11.186	TRUE	0.001	7.523	TRUE
	SIGLEC6	3.024	5.338	4.557	7.462	0.000	21.672	TRUE	0.007	4.357	TRUE	0.000	7.490	TRUE
	CD274/PDL1	2.500	4.305	3.073	4.581	0.001	4.230	TRUE	0.609	1.211	FALSE	0.009	2.844	TRUE
	PDCD1/PD1	1.362	1.656	2.073	3.229	0.009	3.650	TRUE	0.026	2.976	TRUE	0.095	2.228	FALSE
Cytokine/Recptors	IL6	4.174	2.488	2.865	1.860	0.000	-4.972	TRUE	0.146	-1.545	FALSE	0.024	-2.007	TRUE
	IL10	1.449	2.037	1.680	3.709	0.002	4.790	TRUE	0.015	3.187	TRUE	0.004	4.082	TRUE
	LTB	7.513	7.306	7.775	5.674	0.004	-3.578	TRUE	0.010	-3.098	TRUE	0.001	-4.291	TRUE
	TFEC	1.356	5.836	5.116	7.858	0.000	90.678	TRUE	0.003	4.061	TRUE	0.000	6.691	TRUE
	IL2RA	4.204	5.617	6.246	2.777	0.001	-2.688	TRUE	0.000	-7.159	TRUE	0.000	-11.070	TRUE
	IL2RB	2.197	3.887	3.323	5.784	0.000	12.017	TRUE	0.002	3.724	TRUE	0.000	5.506	TRUE
	IL4R	10.764	6.959	8.801	6.986	0.000	-13.717	TRUE	0.969	1.019	FALSE	0.015	-3.518	TRUE
	IL13RA1	8.472	7.544	8.768	5.414	0.000	-8.329	TRUE	0.000	-4.376	TRUE	0.000	-10.221	TRUE
	TNFRSF1B/TNFR2	6.254	7.662	6.738	10.095	0.000	14.327	TRUE	0.000	5.399	TRUE	0.000	10.245	TRUE
	TNFRSF19/TROY	3.057	3.065	2.142	4.683	0.037	3.087	TRUE	0.038	3.070	TRUE	0.002	5.822	TRUE
Differentiation	AICDA/AID	1.755	3.142	1.848	4.010	0.000	4.772	TRUE	0.125	1.824	FALSE	0.001	4.476	TRUE
	BCL6	8.146	5.763	6.736	8.320	0.610	1.128	FALSE	0.000	5.884	TRUE	0.000	2.996	TRUE
Fc Receptors	FCRL3	10.063	8.151	8.336	11.098	0.231	2.048	FALSE	0.002	7.711	TRUE	0.003	6.781	TRUE
	FCRL4	1.553	1.353	1.227	2.941	0.007	2.616	TRUE	0.003	3.006	TRUE	0.001	3.279	TRUE
	FCGR2A/CD32A	2.490	3.806	3.738	4.971	0.000	5.583	TRUE	0.025	2.243	TRUE	0.018	2.350	TRUE
	FCGR2C	2.808	4.396	3.993	6.712	0.000	14.974	TRUE	0.001	4.981	TRUE	0.000	6.583	TRUE
Migration	ITGB2	8.166	7.631	7.657	9.486	0.012	2.498	TRUE	0.001	3.618	TRUE	0.001	3.554	TRUE
	ITGAM/CD11b	5.582	8.698	8.101	7.495	0.000	3.768	TRUE	0.005	-2.302	TRUE	0.132	-1.522	FALSE
	ITGAD	1.915	3.277	2.142	5.173	0.004	9.564	TRUE	0.080	3.721	FALSE	0.007	8.172	TRUE
	CCR7	9.807	7.741	9.182	5.551	0.000	-19.115	TRUE	0.016	-4.564	TRUE	0.000	-12.395	TRUE
	CXCR4	12.122	10.688	11.580	10.146	0.009	-3.933	TRUE	0.450	-1.456	FALSE	0.052	-2.701	FALSE
	CXCR5	8.646	6.689	7.636	3.781	0.000	-29.147	TRUE	0.000	-7.506	TRUE	0.000	-14.472	TRUE
	CCR9	2.700	2.510	3.397	2.735	0.930	1.024	FALSE	0.573	1.168	FALSE	0.103	-1.582	FALSE
	CCR10	1.510	2.201	1.504	1.991	0.451	1.395	FALSE	0.741	-1.157	FALSE	0.445	1.402	FALSE
	CCR6	7.642	6.470	7.251	6.542	0.016	-2.143	TRUE	0.868	1.051	FALSE	0.109	-1.635	FALSE
	CCR2	2.378	2.701	2.613	2.471	0.877	1.067	FALSE	0.703	-1.173	FALSE	0.813	-1.104	FALSE
	CCR5	0.873	1.093	0.751	2.435	0.128	2.952	FALSE	0.189	2.534	FALSE	0.102	3.213	FALSE
	CCR1	1.756	2.014	2.186	1.702	0.849	-1.038	FALSE	0.279	-1.242	FALSE	0.099	-1.399	FALSE
	CXCR3	2.198	3.674	4.264	4.436	0.000	4.716	TRUE	0.049	1.695	FALSE	0.645	1.127	FALSE
Transcription factors/regulators	BCL11B	1.927	3.259	2.247	5.187	0.005	9.584	TRUE	0.086	3.806	FALSE	0.011	7.678	TRUE
	SOX5	1.800	3.975	1.344	7.183	0.000	41.746	TRUE	0.000	9.245	TRUE	0.000	57.266	TRUE
	FOXP4	6.019	5.596	5.672	7.541	0.000	2.871	TRUE	0.000	3.849	TRUE	0.000	3.652	TRUE
	TOX	1.574	7.317	6.422	8.565	0.000	127.206	TRUE	0.022	2.376	TRUE	0.000	4.420	TRUE
Signaling	LCK	5.476	5.443	5.812	2.839	0.000	-6.223	TRUE	0.000	-6.080	TRUE	0.000	-7.855	TRUE
	PTPRS	4.787	4.677	4.713	7.959	0.000	9.013	TRUE	0.000	9.726	TRUE	0.000	9.485	TRUE
	DUSP4	2.154	5.053	3.052	6.945	0.000	27.701	TRUE	0.125	3.712	FALSE	0.003	14.862	TRUE
	DUSP5	6.928	6.128	6.652	8.807	0.046	3.680	TRUE	0.006	6.407	TRUE	0.024	4.456	TRUE
	FGR	7.907	7.150	6.920	10.430	0.000	5.747	TRUE	0.000	9.712	TRUE	0.000	11.388	TRUE
	PRKCH	2.429	5.143	3.706	6.945	0.000	22.873	TRUE	0.012	3.485	TRUE	0.000	9.437	TRUE
	SLAMF7	2.880	4.605	4.650	6.423	0.000	11.658	TRUE	0.000	3.526	TRUE	0.001	3.419	TRUE
	TCL1A	9.805	3.163	6.446	2.177	0.000	-197.811	TRUE	0.379	-1.981	FALSE	0.001	-19.285	TRUE
Other	TOX2	1.406	3.058	2.821	5.833	0.000	21.511	TRUE	0.001	6.845	TRUE	0.001	8.064	TRUE
	GAS7	4.112	5.708	4.644	8.098	0.000	15.840	TRUE	0.000	5.241	TRUE	0.000	10.958	TRUE
	SRGN	5.526	6.282	6.816	8.161	0.000	6.214	TRUE	0.001	3.678	TRUE	0.015	2.541	TRUE
	MMP11	4.039	4.221	3.878	2.458	0.009	-2.992	TRUE	0.004	-3.395	TRUE	0.018	-2.677	TRUE
	MS4A7	5.540	4.863	5.238	7.265	0.008	3.305	TRUE	0.001	5.285	TRUE	0.002	4.074	TRUE
	NKG7	0.949	2.020	2.168	5.856	0.000	30.008	TRUE	0.000	14.284	TRUE	0.000	12.895	TRUE
	CD5	3.859	3.413	3.570	3.369	0.363	-1.405	FALSE	0.934	-1.031	FALSE	0.707	-1.150	FALSE
	BMP6	3.105	3.013	3.691	3.691	0.252	1.501	FALSE	0.186	1.600	FALSE	0.999	-1.000	FALSE
	EMP3	7.534	7.441	7.707	8.763	0.013	2.343	TRUE	0.008	2.500	TRUE	0.029	2.080	TRUE
	S100A4	3.411	4.894	4.620	4.876	0.002	2.761	TRUE	0.966	-1.013	FALSE	0.548	1.194	FALSE

Genes with significant change are indicated (FC>2, p<0.05)

Naïve B cells: CD19^{+} CD11c^{-} IgD^{+} CD38^{int}

Memory B cells: CD19^{+} CD11c^{-} IgD^{-} CD38^{-}

Memory B cells: CD19^{+} CD11c^{-} IgD^{-} CD38^{int}

CD11c^{hi} B cells: CD19^{+} CD11c^{+} IgD^{-} CD38^{-}

Supplementary note 3. Healthy Donor data set

HC		Average expression				CD11c^{hi} vs Naïve			CD11^{hi} vs IgD-CD38- memory			CD11chi vs IgD^{-}CD38^{int} memory		
Category	Symbol	IgD^{+} CD38^{int} naïve	IgD^{-} CD38^{-} memory	IgD^{-} CD38^{int} memory	IgD- CD38- CD11c^{hi}	P value	Fold Change	Significance	P value	Fold Change	Significance	P value	Fold Change	Significance
CD11c^{hi} Phenotype	CD19	7.3297	8.2148	6.6124	8.4178	0.0855	2.2828	FALSE	0.7421	1.1511	FALSE	0.0062	3.4952	TRUE
	ITGAX/CD11c	5.7652	6.3613	6.8261	8.2471	0.0032	5.5866	TRUE	0.0207	3.6957	TRUE	0.0752	2.6778	FALSE
	TBX21/T-bet	5.2297	4.5343	5.2101	6.4205	0.056	2.2828	FALSE	0.0038	3.6967	TRUE	0.0523	2.314	FALSE
	FCRL5	7.123	7.4559	6.7764	9.1688	0.0045	4.1292	TRUE	0.0151	3.2784	TRUE	0.0012	5.2504	TRUE
	FCGR2B/CD32B	5.9475	7.0705	5.5351	6.9784	0.0939	-2.0434	FALSE	0.8781	-1.0659	FALSE	0.0218	2.7195	TRUE
	CD27	3.5463	5.1974	3.5229	4.2571	0.117	1.6368	FALSE	0.0412	-1.9189	TRUE	0.1059	1.6635	FALSE
	CR2/CD21	7.7287	6.9155	7.1035	6.5832	0.1925	-2.2123	FALSE	0.7014	-1.2591	FALSE	0.5491	-1.4343	FALSE
	FCER2/CD23	6.8978	5.9168	5.7308	5.6049	0.038	-2.4502	TRUE	0.6038	-1.2413	FALSE	0.8336	-1.0913	FALSE
	CD24	6.9628	7.8466	6.404	6.6906	0.6199	-1.2076	FALSE	0.042	-2.2283	TRUE	0.6015	1.2198	FALSE
	CD38	4.8015	3.8318	4.5284	3.9155	0.0423	-1.8481	TRUE	0.8421	1.0598	FALSE	0.1523	-1.5293	FALSE
	CD40	6.8878	8.0795	6.1295	6.9943	0.8564	1.0766	FALSE	0.0732	-2.1217	FALSE	0.1492	1.821	FALSE
Activation	CD72	7.5446	6.7951	6.3872	7.717	0.7896	1.1269	FALSE	0.1607	1.8946	FALSE	0.0469	2.5138	TRUE
	TLR9	6.8669	7.2932	7.1321	7.5823	0.1517	1.642	FALSE	0.5564	1.2219	FALSE	0.3618	1.3662	FALSE
	CD70	3.2422	4.3562	4.0477	3.3821	0.7696	1.1018	FALSE	0.0488	-1.9644	TRUE	0.1703	-1.5862	FALSE
Co inhibition	LILRB2/CD85a	4.9086	4.0691	5.4453	5.5655	0.4192	1.5767	FALSE	0.0722	2.8214	FALSE	0.8818	1.0869	FALSE
	SIGLEC6	4.0143	5.6155	4.7533	6.5178	0.0018	5.6705	TRUE	0.2242	1.869	FALSE	0.0217	3.3975	TRUE
	CD274/PDL1	4.8953	4.7816	5.5762	5.2107	0.5595	1.2443	FALSE	0.4283	1.3463	FALSE	0.4991	-1.2884	FALSE
	PDCD1/PD1	4.302	4.3498	4.9344	3.9276	0.5805	-1.2963	FALSE	0.5335	-1.3399	FALSE	0.1438	-2.0095	FALSE
Cytokine/Recptors	IL6	3.8304	3.7307	3.7911	3.7854	0.9155	-1.0316	FALSE	0.8972	1.0387	FALSE	0.9893	-1.0039	FALSE
	IL10	2.4403	2.977	3.1399	3.0205	0.3773	1.4951	FALSE	0.9468	1.0306	FALSE	0.855	-1.0862	FALSE
	LTB	6.4896	8.4173	5.7776	6.9411	0.4495	1.3675	FALSE	0.0181	-2.7822	TRUE	0.0579	2.24	FALSE
	TFEC	4.7089	4.2907	4.9002	5.2526	0.3833	1.4577	FALSE	0.1282	1.948	FALSE	0.5705	1.2767	FALSE
	IL2RA	4.9607	5.5224	5.5117	4.9373	0.9496	-1.0164	FALSE	0.1221	-1.5002	FALSE	0.1288	-1.4891	FALSE
	IL2RB	4.4551	5.1071	5.1084	5.9553	0.0116	2.8289	TRUE	0.1381	1.8003	FALSE	0.1386	1.7987	FALSE
	IL4R	8.108	6.6603	6.5264	6.3788	0.0193	-3.3155	TRUE	0.6895	-1.2155	FALSE	0.8338	-1.1078	FALSE
	IL13RA1	7.3411	6.8358	7.208	6.4116	0.0536	-1.9046	FALSE	0.0953	-1.3419	FALSE	0.3658	-1.7368	FALSE
	TNFRSF1B/TNFR2	7.5854	8.4617	7.2975	9.1421	0.0536	2.9418	TRUE	0.3658	1.6026	FALSE	0.0953	3.5915	TRUE
	TNFRSF19/TROY	5.69	5.3656	6.6601	6.032	0.0064	1.2675	FALSE	0.2091	1.5871	FALSE	0.0016	-1.5456	FALSE
Differentiation	AICDA/AID	4.4483	3.9597	5.2283	4.8091	0.6492	1.2841	FALSE	0.3778	1.8018	FALSE	0.4054	-1.3372	FALSE
	BCL6	5.8718	5.5592	6.4567	5.5458	0.5167	-1.4345	FALSE	0.1331	1.4255	FALSE	0.4517	-1.0225	FALSE
Fc Receptors	FCRL3	7.1332	7.6139	6.243	9.1757	0.1333	4.1196	TRUE	0.14	2.9522	FALSE	0.9246	7.6352	TRUE
	FCRL4	2.8306	3.8212	4.2381	4.0753	0.0222	2.3697	TRUE	0.0748	1.1926	FALSE	0.0017	-1.1195	FALSE
	FCGR2A/CD32A	5.1371	4.8257	5.6877	5.1143	0.0148	-1.0159	FALSE	0.6004	1.2215	FALSE	0.7367	-1.488	FALSE
	FCGR2C	4.7413	5.1205	5.3656	5.7372	0.9634	1.9943	FALSE	0.5614	1.5333	FALSE	0.2528	1.2937	FALSE
Migration	ITGB2	7.9487	8.2833	7.437	9.033	0.1047	2.1204	TRUE	0.3081	1.6815	FALSE	0.5367	3.0231	TRUE
	ITGAM/CD11b	6.3836	7.2465	6.9334	7.3078	0.0363	1.8976	TRUE	0.1398	1.0434	FALSE	0.0031	1.2963	FALSE
	ITGAD	5.3079	5.5433	6.6745	5.8675	0.0249	1.4738	FALSE	0.8763	1.2519	FALSE	0.3453	-1.7497	FALSE
	CCR7	6.8566	7.7085	5.5375	6.2028	0.5956	-1.5733	FALSE	0.7581	-2.8397	FALSE	0.4452	1.5858	FALSE
	CXCR4	9.2129	9.2745	7.9979	7.8186	0.4493	-	FALSE	0.088	-2.7432	TRUE	0.4415	-1.1323	FALSE
	CXCR5	6.9184	7.4476	6.3648	6.574	0.0583	-1.2696	FALSE	0.0486	-1.8323	FALSE	0.8016	1.156	FALSE
	CCR9	3.0761	2.893	3.463	3.4032	0.5327	1.2544	FALSE	0.1202	1.4242	FALSE	0.7042	-1.0423	FALSE
	CCR10	3.0761	2.893	3.463	3.4032	0.4126	-1.145	FALSE	0.205	1.8894	FALSE	0.8801	-1.2246	FALSE
	CCR6	6.7953	6.5653	6.403	6.5039	0.7583	-1.2239	FALSE	0.1553	-1.0435	FALSE	0.6456	1.0725	FALSE
	CCR2	2.6932	2.9931	3.8001	3.4205	0.5014	1.6555	FALSE	0.8869	1.3448	FALSE	0.8153	-1.3009	FALSE
	CCR5	2.2025	1.8988	2.8582	2.1039	0.2332	-1.0707	FALSE	0.4799	1.1528	FALSE	0.5301	-1.6868	FALSE
	CCR1	1.9382	2.4819	2.4678	2.6092	0.9218	1.5923	TRUE	0.8382	1.0923	FALSE	0.4549	1.103	FALSE
	CXCR3	4.396	5.2748	5.0411	5.378	0.025	1.9753	TRUE	0.6567	1.0742	FALSE	0.6217	1.263	FALSE
Transcription factors/regulators	BCL11B	5.6562	5.9942	6.9533	6.4554	0.0129	1.7402	FALSE	0.7823	1.3766	FALSE	0.3702	-1.4122	FALSE
	SOX5	4.1257	4.594	5.0485	5.8007	0.4664	3.1931	TRUE	0.6733	2.308	FALSE	0.6491	1.6843	FALSE
	FOXP4	6.7264	6.5593	6.527	6.9896	0.0394	1.2001	FALSE	0.1308	1.3475	FALSE	0.3403	1.378	FALSE
	TOX	4.0582	5.3693	5.0352	6.6769	0.4265	1.0523	FALSE	0.1977	1.5618	FALSE	0.1671	-1.7673	FALSE
Signaling	LCK	6.7535	6.3911	6.6275	6.0407	0.9221	-1.639	FALSE	0.3949	-1.2749	FALSE	0.2791	-1.5018	FALSE
	PTPRS	7.111	7.0018	8.0801	7.729	0.1681	1.5348	FALSE	0.4926	1.6555	FALSE	0.2541	-1.2755	FALSE
	DUSP4	5.795	6.4181	7.2127	6.615	0.3702	1.7654	FALSE	0.293	1.1462	FALSE	0.609	-1.5133	FALSE
	DUSP5	6.0673	6.08	6.0608	6.3252	0.4983	1.1957	FALSE	0.8703	1.1853	FALSE	0.6209	1.2011	FALSE
	FGR	8.0799	8.1965	7.3287	9.7556	0.7771	3.1948	TRUE	0.7877	2.9467	TRUE	0.7716	5.3773	TRUE
	PRKCH	4.8897	4.2748	5.4101	5.7781	0.0082	1.851	FALSE	0.0132	2.8348	TRUE	0.0003	1.2905	FALSE
	SLAMF7	4.2401	4.4991	4.667	4.8208	0.1977	1.4956	FALSE	0.0336	1.2498	FALSE	0.5891	1.1125	FALSE
	TCL1A	8.6007	6.0194	6.0818	5.3245	0.207	-9.6885	TRUE	0.4803	-1.6188	FALSE	0.735	-1.6904	FALSE
Other	TOX2	4.6405	4.6127	5.9429	5.7026	0.006	2.0879	FALSE	0.5342	2.1285	FALSE	0.4984	-1.1813	FALSE
	GAS7	6.7571	6.2148	7.1698	7.5243	0.1747	1.702	FALSE	0.1641	2.4786	TRUE	0.7551	1.2785	FALSE
	SRGN	5.0042	5.096	5.0285	5.861	0.2132	1.8111	FALSE	0.0382	1.6994	FALSE	0.5609	1.7808	FALSE
	MMP11	6.0768	6.0094	6.3687	5.5715	0.1089	-1.4195	FALSE	0.1505	-1.3546	FALSE	0.1189	-1.7378	FALSE
	MS4A7	5.1369	5.3439	5.2691	6.0456	0.3792	1.8774	FALSE	0.4452	1.6264	FALSE	0.1696	1.7129	FALSE
	NKG7	3.4617	3.7282	4.0931	4.8871	0.1458	2.6859	FALSE	0.2578	2.2328	FALSE	0.2116	1.7338	FALSE
	CD5	5.2237	4.6384	5.317	4.6344	0.0527	-1.5045	FALSE	0.1112	-1.0028	FALSE	0.2695	-1.605	FALSE
	BMP6	4.7703	5.0462	5.6551	4.5457	0.2756	-1.1684	FALSE	0.9941	-1.4147	FALSE	0.2083	-2.1575	TRUE
	EMP3	6.2303	8.2736	6.0055	7.9859	0.6571	3.3767	TRUE	0.3259	-1.2208	FALSE	0.0348	3.9459	TRUE
	S100A4	4.1234	5.5075	4.6153	4.7041	0.0007	1.4956	FALSE	0.5371	-1.7453	FALSE	0.0002	1.0635	FALSE

Genes with significant change are indicated (FC>2, p<0.05)

Naïve: CD19^{+} CD11c^{-} IgD^{+} CD38^{int}

Memory: CD19^{+} CD11c^{-} IgD^{-} CD38^{-}

Memory: CD19^{+} CD11c^{-} IgD^{-} CD38^{int}

CD11c^{hi}: CD19^{+} CD11c^{+} IgD^{-} CD38^{-}
